# The ability to detach from biofilms in the lung airways prior to transmission to another host is associated with the infectious phenotype of *Mycobacterium abscessus*

**DOI:** 10.3389/fimmu.2025.1508584

**Published:** 2025-03-07

**Authors:** Bailey Keefe, Amy Leestemaker-Palmer, Luiz E. Bermudez

**Affiliations:** ^1^ Department of Biomedical Sciences, Carlson College of Veterinary Medicine, Oregon State University, Corvallis, OR, United States; ^2^ Department of Microbiology, College of Sciences, Oregon State University, Corvallis, OR, United States

**Keywords:** *mycobacterium abscessus*, biofilm, detachment, infectious phenotype, host bacterial surface, macrophages, uptake

## Abstract

**Introduction:**

*Mycobacterium abscessus* is a pathogen recently associated with patients with chronic lung conditions such as bronchiectasis and cystic fibrosis. *M. abscessus* is an environmental bacterium but recent evidence suggests that the pathogen is also transmitted from host-to-host. Because *M. abscessus* is known to form biofilms on the respiratory mucosa the release of bacteria from the biofilm becomes an important aspect on the transmission of the infection.

**Methods:**

A biofilm releasing system was established. A transposon library of *M. abscessus* was then screened to identify genes associated with the release from biofilms.

**Results:**

Several enzymes and genes of unidentified function were linked with the ability to detach from the biofilm. It was also shown that detached bacteria were increased capable of establish a new biofilm, attach to epithelial cells, and infect macrophages. To determine the surface molecules linked with the ability to infect new hosts, a surface proteomic was performed, showing that detaching bacteria express many proteins do not present in biofilm bacteria.

**Discussion:**

Detached *M. abscessus*, one of the possible infectious phenotypes, contains specific proteins and lipids in the surface that facilitate the infection of new hosts. In addition, we identified many small proteins that have the likelihood to be associated with the release of the biofilm bacteria.

## Introduction

Pulmonary infections caused by *Mycobacterium abscessus* have been increasingly identified in individuals with chronic lung pathologies, such as emphysema, bronchiectasis, and cystic fibrosis (1–3). All three subspecies of *M. abscessus*, i.e., *M. abscessus* subsp. *abscessus*, *M. abscessus* subsp. *Bolletii*, and *M. abscessus* subsp. *masssiliense*, are resistant to the majority of available antibiotics, making the treatment of patients quite challenging (3, 4).

There is evidence that *M. abscessus* has evolved recently in order to adapt to the airway environment of humans harboring the risk factors (5, 6). Not only is the infection becoming more common, but additional evidence of genome evolution has also emerged (7, 8). A few clones of the pathogen have been isolated from infections in the respiratory tracts of patients, which suggests rapid evolution allowing for adaptation to the environment.

One of the strategies by which environmental bacteria adapt to mammalian host infections is the inherent ability to form a biofilm (BF). Studies and clinical experience have shown that *M. abscessus* uses that resource to initiate and maintain a niche or niches on the airway mucosal surface in patients with chronic lung pathologies (9, 10). To achieve this, the bacterium rapidly needs to establish and develop the biofilm structure before being challenged by the host immune cells or anti-bacterial products secreted by the host cells. We have previously described how *M. abscessus* responds to the environmental conditions of the airways with the efficient formation of biofilm (9). It is apparent that the bacterium uses the magnesium concentration in the mucus of cystic fibrosis patients to quickly adhere to the respiratory mucosa and develop a robust biofilm (9–11). It is also puzzling that the pathogen uses the host DNA existing in the airways as a carbon source, which apparently also has a role in the transition from the planktonic to the biofilm phenotype (12).

Considering all the accumulated pieces of information and adding the recent evidence that *M. abscessus* can be also transmitted among cystic fibrosis patients, in many cases, within the shared environment of a clinic waiting room or other facilities (7), we hypothesized the bacteria being released from airway biofilms would most likely represent an infectious phenotype of the pathogen. In fact, the ability to detach from biofilm can be linked with additional site seeding in the airways which would have implications in the extension of the disease. Understanding the pathogenic mechanisms associated with the infection will allow for the development of prevention measures or potentially the elimination of the disease in the at-risk population.

To address the question, we established an *in vitro* set of assays to identify detached organisms and the genes associated with the ability. Our findings identified many genetic components pointing to an association with genes and encoded proteins linked to surface structures and the transport mechanisms that exist to export these proteins to the bacterial surface. Further studies determined that some of these proteins participate in the attachment to bronchial epithelial cells.

## Materials and methods

### Bacterial strains and growth

*Mycobacterium abscessus* subsp. *abscessus* strain 19977 was obtained from the American Type Culture Collection (ATCC). *M. abscessus* subsp. *abscessus* strains 00103 and 01715 (obtained from patients with cystic fibrosis) were a gift from Jerry Nick and Charles Daley from the National Jewish Health Hospital. All three strains were of the *in vitro* smooth phenotype. Unless otherwise noted, bacteria for assays were grown and maintained on Middlebrook 7H10 agar supplemented with 10% oleic acid, albumin, dextrose, and catalase (OADC; Hardy Diagnostics), referred to as 7H10. For liquid cultures, bacteria were grown in 7H9 broth supplemented with Tween-80 and OADC, referred to as 7H9. All the cultures were grown at 37°C for 3 to 5 days. The bacteria used in the assays were of the planktonic phenotype initially. Every assay was controlled by microscopic observation so that the inoculum completely dispersed.

### Host cell tissue culture

Human monocyte THP-1 (TIB-202) and HEp-2 (CCL-23) pharyngeal epithelial cells were obtained from the ATCC and grown in RMPI-1640 (RPMI) and DMEM sterile media containing L-glutamine, 25 mM HEPES (Corning Life Sciences), and 10% fetal bovine serum (FBS, Gemini Bio-products, Sacramento, CA). Both cell types were obtained from the ATCC and maintained at 37°C with 5% CO_2_.

### Generation of secondary biofilms in either HBSS or SCFM media

*M. abscessus* biofilms were formed by generating a turbid suspension of the bacteria in HBSS. Turbidity was measured via O.D. using an EPOCH spectrophotometer (Biotex) at 595nm to obtain a concentration of 3 x 10^8^ CFU/mL. SCFM media were prepared as described previously (9, 13) and utilized as a biofilm formation condition in tandem with HBSS biofilm formation. For the biofilms formed in SCFM, bacteria were diluted to obtain 1 x 10^6^ CFU/200 µL and biofilms formed in HBSS were diluted to obtain 1 x 10^7^ CFU/200 µL. Secondary biofilms were established by collecting the supernatant from primary biofilms during the replicative stage (day 2) or non-replicative stage (day 5) of biofilm formation and then placed in fresh SCFM or HBSS. SCFM and HBSS were replaced with fresh media every 24 h so that the supernatants mainly contained released bacteria during biofilm formation. Secondary biofilms were established for 7 and 14 days. The biomass of the primary and secondary biofilms was quantified using the crystal violet assay (14) and absorbance was measured at 550 nm. CFU quantitation for the biofilms was obtained by mechanically disrupting the biofilms via pipetting, followed by serial dilutions and plating on 7H10 agar. Planktonic bacteria at 1 x 10^5^, 1 x 10^6^, and 1 x 10^7^ CFU/mL served as biofilm controls.

### Generation of the *M. abscessus* 19977 mycomarT7 transposon library

The *M. abscessus* transposon library was generated as described previously (15). MycomarT7 (mmT7) is a temperature-dependent transposon-containing phagemid and was kindly provided by Eric Rubin (Harvard T.H. Chan School of Public Health, Boston, MA). MmT7 was propagated and titers were generated using *M. smegmatis* strain mc^2^ 155 as described previously (15). *M. abscessus* was grown in 7H9 broth supplemented with 10% OADC and 0.1% Tween-80 at 37°C in a shaking incubator before transduction. Bacteria were then pelleted and washed with MP buffer (150 mM NaCl, 50 mM Tris-HCl, 10 mM Mg_2_SO_4_, 2 mM CaCl_2_) twice. Washed bacteria were resuspended in MP and buffered and infected with mmT7 at an MOI of 2. The transduction commenced at 37°C for 4 hours with intermittent mixing. Aliquots were plated on 7H10 containing 400 µg/mL kanamycin to obtain individual transposon mutants. A selection of mutants was screened for the presence of mmT7 by amplifying the kanamycin resistance gene with PCR.

### Screening the *M. abscessus* transposon library for deficiency in secondary biofilm formation

The SCFM and HBSS biofilm conditions were screened to identify mutants unable to release bacteria to form secondary biofilms. For the SCFM biofilms, individual mmt7 mutants were selected and cultured in 7H9 broth containing 400 µg/mL kanamycin for 4 days at 37°C in a shaking incubator using a 96-deep-well plate format. After 4 days, bacteria were pelleted and resuspended in HBSS. Fresh SCFM was aliquoted into a flat bottom 96-well plate, and 50 µL of each mutant suspension was added to the plate. Biofilms were formed for 7 days in the dark at 25°C with supernatant replacement after 2 days and then the resulting supernatant was carefully removed to form a secondary biofilm. These secondary biofilms were allowed to form for another 7 days and then the O.D. of the supernatant was measured along with the biomass of the biofilm using the crystal violet assay. Mutants were selected based on diminished secondary biofilm biomass and low O.D. in the corresponding supernatant compared to the wild-type.

For HBSS biofilms, individual mmt7 mutant colonies were added to a 48-well plate containing 7H9 broth media and allowed to grow for 3-4 days. Once mutants had grown to 3 x 10^8^ CFU/mL, 100 µL of the bacteria was placed in 900 µL of HBSS in a fresh 48-well plate to form biofilm for 7 days in the dark at 37°C with supernatant replacement after 2 days. After 7 days, the supernatant was carefully removed without disturbing the transposon mutant biofilm and placed in another fresh well containing HBSS. The supernatant of each of these mutants was allowed to form biofilm for another 7 days, and the O.D. at 595 nm was measured, along with a visual inspection for opaqueness. Wells that matched the O.D. of HBSS alone or had a low O.D. were considered deficient in release or secondary biofilm formation. These mutants were selected for Sanger sequencing using an ABI 3730 capillary sequence machine within the Center for Quantitative Life Sciences (CQLS) at Oregon State University.

### Identifying transposon insertion location within *M. abscessus* mutants

Transposon mutants were reconfirmed in duplicate before sequencing as previously described (9). Briefly, mutants selected from the biofilm detachment screen were sequenced utilizing a previously reported LM-PCR assay with some changes (16). Cells were lysed using 1-mm-diameter glass beads in diH_2_O via mechanical homogenization. Lysates were centrifuged for 1 minute at 21,000 x *g* to pellet cell debris. DNA was purified from collected supernatant using a DNA clean and concentrate kit (Zymo Research) following the manufacturer’s protocol. Furthermore, 150 ng of purified DNA was utilized for single digestion with SalI (Thermo fast-digest enzyme) for 30 minutes at 37°C. LM-PCR adapter oligos were generated for SalI (Salgd+Salpt, see **Table 1**) by adding 45 mM of each oligonucleotide to 1x Taq DNase buffer plus MgCl_2_ and ligating by decreasing the temperature from 80°C to 4°C over an hour. After digestion, DNA for each mutant was ligated with the adapter oligos using T4 DNase ligase. The ligated product was used as the template in the LM-PCR reaction. The LM-PCR reaction utilized Gold 360 MasterMix (Applied Biosystems) and was performed using 97°C for 7 min, 40 cycles of 97°C for 30 s, 58°C for 1 min, and 72°C for 1 min 45 s, and then a final step of 72°C for 10 min. PCR products were visualized using gel electrophoresis and ethidium bromide. The bands of interest were excised and purified using a gel extraction kit (Thermo) and sequencing at the Center for Quantitative Life Sciences (CQLS) at Oregon State University. Sequencing results were blasted in the NCBI against *M. abscessus* to identify disrupted genes.

**Table 1:** Primers used in the PCR, LM-PCR, and complementation assays.

Primer	Sequence (5’ to 3’)
Saldg (for both adapters)	TAGCTTATTCCTCAAGGCACGAGC
Salpt (for SalI adapter)	TCGAGCTCGTGC
pSalg R (for LMPCR reaction)	GCTTATTCCTCAAGGCACGA
pMyco F1 (for LMPCR reaction)	CCGGGGACTTATCAGCCAAC
pMyco F2 (for LMPCR reaction)	ACCCGTGATATTGCTGAAGAG
MAB_4706ccompF	AAAAAAtctagaGCCACAATCCGAAATTTGTT
MAB_4706ccompR	TTTTTTaagcttTTTGGGCGACTAGGAAGCTA
Kan F	ATGACTGGGCACAACAGAC
Kan R	TCGTCAAGAAGGCGATAGA

### Complementation of *M. abscessus* mutant MAB_4706c

The MAB_4706c gene with native promoters was complemented by the use of the integrative pMV306 plasmid as previously described (17) with some changes. First, an apramycin-resistance gene was cloned into pMV306 to generate the pMV306-Apr plasmid. Genes were cloned into pMV306-Apr using primers designed for these specific regions. This product was then transformed into electrocompetent DH10β *E. coli* and grown on LB containing 400 µg/mL kanamycin and apramycin. Positive colonies were used for colony PCR to confirm apramycin resistance and gene of interest ligation. These constructs were extracted using a Qiagen Mini Prep kit as per the manufacturer’s protocol. Electrocompetent *M. abscessus* mutant cells were prepared by washing plate-grown bacteria four times via centrifugation at 2000 x *g* at 4°C for 10 minutes in an ice-cold 10% glycerol and 0.1% Tween-80 solution. Cells were stored at -80°C in 10% glycerol until use. Plasmids were electroporated into *M. abscessus* competent cells using a 0.2cm cuvette (BioRad) at 2500 V, 1000Ω, and 25µF. Bacteria were recovered in 7H9 for 2 to 3 hours and plated onto 7H10 containing 400 µg/mL of kanamycin and apramycin and positive colonies were screened via PCR as described above.

### Addition of monocytes to *M. abscessus* biofilms

Biofilms were established for 7 days in either HBSS or SCFM as described above. THP-1 monocytes were stimulated with 100 ng/mL of IFN-γ for 24 h prior to biofilm contact. After biofilm maturation, supernatants were removed and replaced with stimulated THP-1 monocytes (1x10^5^ cells/well) in RPMI media. The biofilms were mechanically lysed at days 1, 2, and 3 post THP-1 addition to determine macrophage activity against these biofilms as quantified by CFUs. Activated THP-1 cells were also added during the replicative stage and non-replicative stage of biofilm formation by replacing supernatants with RMPI media followed by CFU determination at days 1 and 3 post contact with monocytes via serial dilution.

### Infection and binding of host cells with *M. abscessus* biofilm phenotypes

THP-1 cells were seeded at 3 x 10^5^ cells/500 µL of RPMI supplemented with 10% FBS containing 50 ng/mL phorbol 12-myristate 13-acetate (PMA) and incubated at 37°C. After 24 hours, PMA was removed and replaced with fresh complete media and incubated overnight at 37°C. Biofilms were formed as previously described in either HBSS or SCFM. At day 7, the biofilms were disrupted to release bacteria and then THP-1 macrophages were infected with primary biofilm or secondary biofilm bacteria for 1 hour, including a 10-min synchronization step at 150 x *g*. To kill extracellular bacteria, 200 µg/mL of amikacin was added to the monolayers for 2 hours. Cells were lysed post antibiotic treatment, at 24 hours, and 72 hours post infection. Lysates were serial diluted and plated on 7H10 agar for intracellular CFU quantification.

HEp-2 cells were seeded with DMEM media containing 10% FBS and allowed to achieve 90% confluency. Cells were then overlayed with ice-cold culture medium containing 10^6^ CFU/mL bacteria to prevent internalization of bacteria by the epithelial cells. Wild-type *M. abscessus* 19977, secondary biofilm mutants, and a complemented strain were utilized for infection. The *M. abscessus* strains were allowed to bind to epithelial cells for 30 min at 4°C. Monolayers were then washed with ice-cold HBSS twice and lysed with 0.01% TritonX-100 and serially diluted on 7H10 agar for CFU determination.

### Isolation of bacterial surface proteins and proteomic identification

To isolate surface proteins from bacteria, we utilized biotinylation followed by streptavidin-bound magnetic bead purification. We compared planktonic bacteria, bacteria with the biofilm phenotype in HBSS (7-day biofilm), bacteria that formed secondary biofilms in HBSS, and bacteria that formed secondary biofilms in SCFM. First, bacteria cells were washed twice with HBSS. After the 2nd wash, bacteria pellets were resuspended in 1 mg Sulfo-NHS-LC-biotin (Thermo Scientific) reconstituted in 1mL HBSS and then incubated for 30 min at 4°C with gentle rotation. Leftover biotin reagent was quenched with 10 mM glycine in HBSS for 10 min at room temperature under gentle rotation. Bacteria cells were washed twice with 1mL HBSS. After washing, the pellets were resuspended with GLIB buffer (10 mM EDTA, 2 mM EGTA, 0.1% tween-20, 6 M guanidinium HCl in PBS, pH 7.2) and then transferred to a 2 mL bead-beating tube with <0.1 mm glass beads.

Bacteria samples were lysed using an Omni Bead Ruptor (Omni Intl.) set to speed 4 with three cycles of 30 s between each cycle samples and were placed on ice to prevent degradation. To clear, the lysate samples were centrifuged for 1 min at room temperature at 21,000 x g. The supernatant was collected and passed through a 0.2 µm syringe filter to remove DNA and any large debris from the protein extract. The supernatant was transferred to a 2 mL protein lo-bind (Eppendorf) tube. Streptavidin Dynabeads (Thermo Scientific) were added to the extract and incubated for 1 hour in a rotary shaker at room temperature. The protein mixture was transferred into a µMacs magnetic column (Miltenyi Biotec). The beads were washed twice with GLIB buffer with an incubation step in between for 5 mins on a rotary shaker. After second GLIB wash, the beads were washed twice with PBS (0.05% tween-20 in PBS, pH 7.2) in a new clean tube with an incubation of 30 min on a rotary shaker at room temperature. Beads containing surface proteins were eluted in resuspension buffer (1% SDS, 10 mM EDTA in H_2_O) and then incubated at 65°C for 10 min to denature proteins from the beads for mass spectrometry.

To reduce the disulfide bonds of the proteins, the samples were incubated at 56°C for 1 hour with 5 mM dithiothreitol (ThermoFisher). The samples were then incubated with 10 mM iodoacetamide (MilliPore Sigma) for 1 hour at room temperature in the dark in order to carbamidomethylate the cysteine residues. The samples were digested overnight at 37°C using Trypsin Gold (Mass Spectrometry Grade, Promega). After digestion, the samples were spun down at 12,000 x g for 30 s to collect the condensate, and the digestion was stopped by the addition of 0.5% (v/v) trifluoroacetic acid. The samples were centrifuged at 12,000 x g for 10 minutes and then transferred to LC vials.

A Waters nanoAcquityTM UPLC system (Waters, Milford, MA) was coupled to an Orbitrap Fusion Lumos mass spectrometer (Thermo Fisher Scientific). Peptides were loaded onto a trap 2G nanoAcquity UPLC Trap Column (180um, 50mm, 5um) at a flow rate of 5 μL/min for 5 min. The results were obtained on a commercially available Acquity UPLC Peptide BEH C18 column (100um, 100mm, 1.7um). The column temperature was maintained at 37°C using the integrated column heater. Solvent A was 0.1% formic acid in LC-MS grade water and solvent B was 0.1% formic acid in LC-MS grade acetonitrile. The separation was performed at a flow rate of 0.5 μL/min, and using linear gradients of 3% to 10% B for 10 min, 10% to 30% B for 10 min, 30% to 70% B for 5 min, 70% to 95% B for 3 min, 95% to 3% B for 4 min, and 95% to 3% B for 3 min. Total method length was 35 min. The outlet of the column was connected to a Thermo Nanospray Flex ion source and +2300V were applied to the needle.

MS1 spectra were acquired at a resolution of 120,000 (at m/z 200) in the Orbitrap using a maximum IT of 50 ms and an automatic gain control (AGC) target value of 2E5. For MS2 spectra, up to 10 peptide precursors were isolated for fragmentation (isolation width of 1.6 Th, maximum IT of 10 ms, and AGC value of 2e4). Precursors were fragmented by HCD using 30% normalized collision energy (NCE) and analyzed in the Orbitrap at a resolution of 30,000. The dynamic exclusion duration of fragmented precursor ions was set to 30 s. Raw files were processed in Thermo Proteome Discoverer 2.3. Precursor ion mass tolerance was set to 5 ppm, while fragment ion mass tolerance was 0.02 Da. The SequestHT search engine was used to search against the Swissprot human and *M. abscessus* protein database. Only b and y ions were considered for peptide spectrum matching. MS1 precursor quantification was used for label-free quantitation of the peptides. Protein abundances were calculated as the sum of the abundances of unique peptides detected.

### Statistics

Statistical analyses were performed using GraphPad Prism 9 software. The comparisons between the treatment groups were analyzed using either t-tests or analysis of variance (ANOVA) with multiple comparisons where appropriate. Specific statistical tests are named in the figure legends in which they were used. A P-value of < 0.05 was considered significant.

## Results

### *M. abscessus* is able to form secondary biofilms

**Figure 1:**
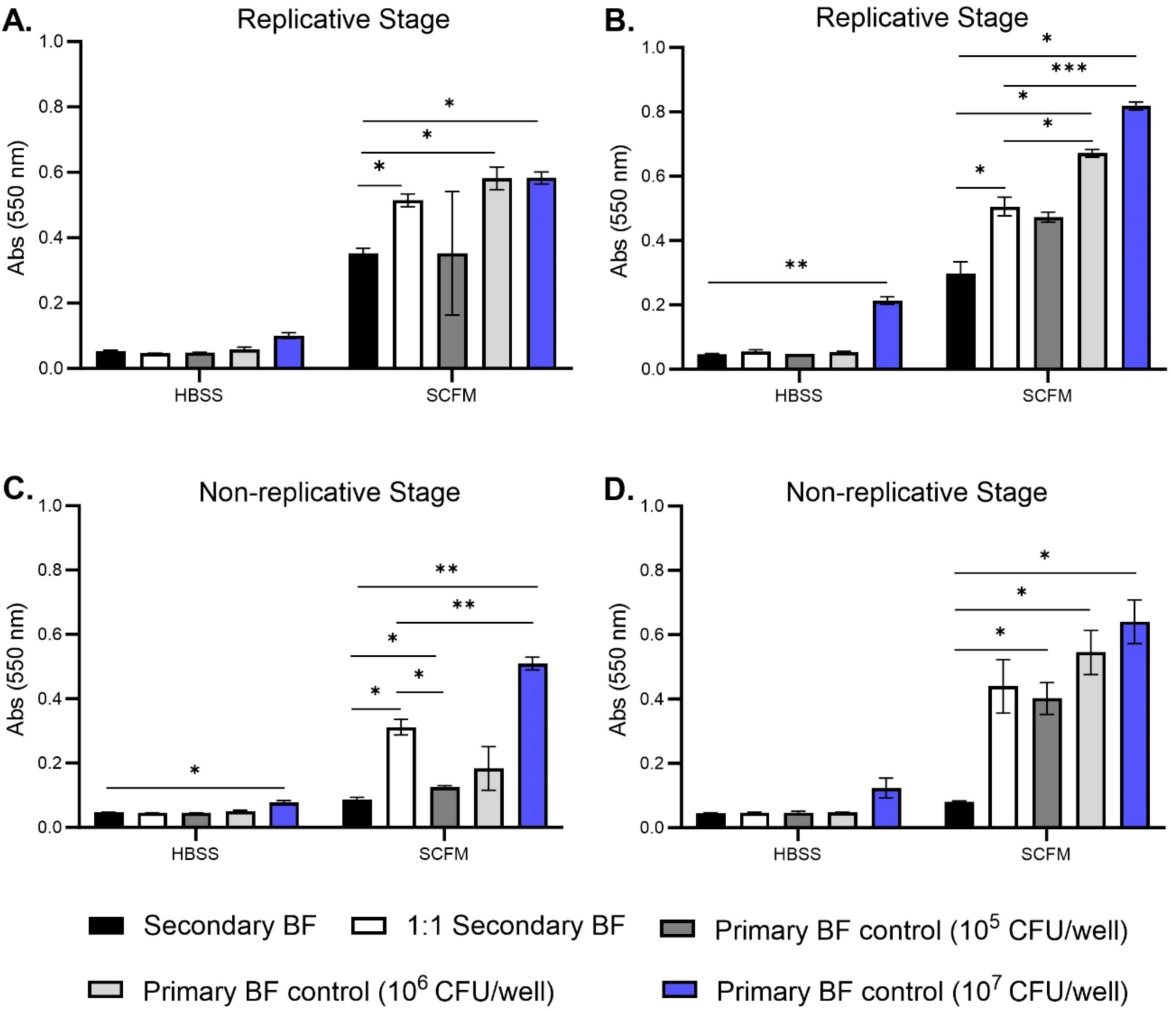
*M. abscessus bacteria released from primary biofilms are capable of establishing secondary biofilms*. *M. abscessus* bacteria released from replicative stage biofilms and non-replicative stage biofilms were utilized to reform secondary biofilms in both HBSS and SCFM conditions. Biomass was measured for replicative stage secondary biofilms at days 7 **(A)** and 14 **(B)**. Biomass was measured for non-replicative stage secondary biofilms at days 7 **(C)** and 14 **(D)** via crystal violet assay. BF, biofilm. The error bars represent the SEM of three biological replicates. Statistical analysis was conducted using Brown–Forsythe and Welch ANOVA with a Dunnett’s T3 multiple comparison test; *** indicates p < 0.0001, ** indicates p < 0.001, * indicates p < 0.05.

A key aspect of the biofilm formation cycle is the bacteria’s ability to detach from the biofilm and establish a secondary or satellite biofilm. Growing evidence has been mounting of patient-to-patient transmission in hosts that have cystic fibrosis, but more information is needed to ascertain this infectious phenotype of *M. abscessus*. Previous work in our lab showed that *M. abscessus* can form robust biofilms in synthetic cystic fibrosis media (SCFM), and specifically that the colony forming units (CFUs) of the biofilm do not increase after day 4, while the matrix biomass continues to increase over time (9). Based on this, the capability of bacteria released from replicative stage biofilms and non-replicative stage biofilms to re-form biofilms was investigated. Supernatants of bacteria forming biofilms were carefully removed from wells on day 2 (replicative stage) and day 5 (non-replicative stage) and utilized to form biofilms. These secondary biofilms were developed in either a 1:1 ratio of supernatant to fresh media or unaltered transferred supernatant (**Figure 1**). Secondary biofilms were established for 7 and 14 days in Hank’s balanced salt solution (HBSS) or SCFM. The biomass was determined at both time points, and matched primary biofilms of wild-type bacteria were included under SCFM and HBSS formation conditions. Released bacteria from both the replicative stage (**Figures 1A, B**) and non-replicative stage biofilms (**Figures 1C, D**) were able to form secondary biofilms, especially when introduced to fresh media. The biomass of the SCFM biofilms formed with non-replicative stage bacteria matched the biomass of wild-type bacteria when added into fresh SCFM at both time points (**Figures 1C, D**). The biomass of the secondary biofilms transferred without fresh media remained significantly lower than the control groups in all conditions. There did not appear to be differences in the biomass of secondary biofilms formed by released bacteria in HBSS compared to wild-type bacteria except at day 14 for the replicative stage supernatants and day 7 for the non-replicative stage supernatants compared to the 10^7^ bacteria control (**Figures 1B, C**, respectively).

**Figure 2:**
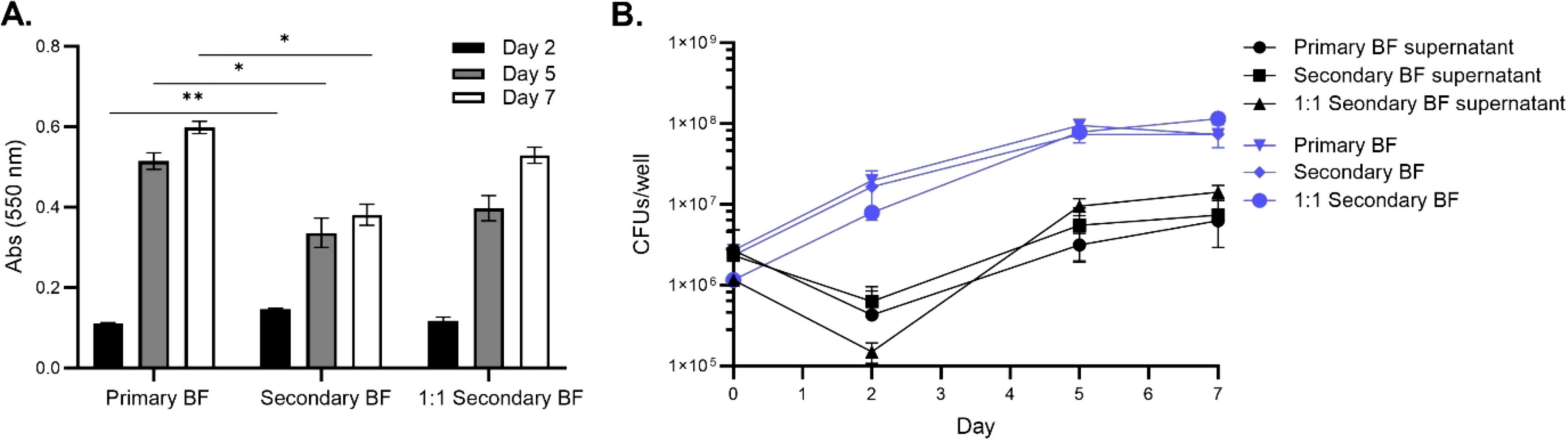
*Secondary biofilms were established similarly to wild-type M. abscessus primary biofilms in SCFM.* The replicative stage supernatants of SCFM primary biofilms were used to form secondary biofilms. The biomass was determined for the primary biofilm, unaltered supernatant forming a secondary biofilm, and a 1:1 ratio of supernatant with fresh SCFM forming a secondary biofilm **(A)**. The CFU/well was determined for both the biofilm and released bacteria in the supernatant **(B)**. For both assays, the timepoints were measured at days 2, 5, and 7 of the formation process. BF, biofilm. The error bars represent the SEM of two biological replicates. Statistical analysis was conducted using Brown–Forsythe and Welch ANOVA with a Dunnett’s T3 multiple comparison test; ** indicates p < 0.001, * indicates p < 0.05.

Next, assays were performed to determine whether the replicative stage biofilm-released bacteria had any advantage in establishing secondary biofilms more quickly than the wild-type bacteria in SCFM conditions. For CFU determination, both the disrupted biofilms and released biofilms into the supernatant were enumerated. Similarly, the replicative stage released bacteria were placed in either a 1:1 ratio of supernatant to fresh SCFM media or unaltered transferred supernatant and the biomass and CFUs were measured at days 2, 5, and 7 during biofilm formation (**Figure 2**). As seen previously, all the conditions established more robust biomass by day 5, with day 7 staining showing the highest biomass [**Figure 2A** (9)]. Unaltered supernatants were significantly increased by day 2; however, by days 5 and 7, the secondary biofilm biomass was less than the primary wild-type biofilms (**Figure 2A**). The secondary biofilms, when given fresh media, had similar biomass accumulation to the wild-type. The CFU/well was obtained for the bacteria in the biofilm or detached phenotypes (**Figure 2B**). Bacteria replication (CFU/mL) in the secondary biofilm conditions increased on day 2 but plateaued by day 5 whereas the supernatant phenotypes dropped on day 2 but then increased by day 5, suggesting the bacteria were switching to biofilm-formation phenotypes between days 0 and 2 but increased biomass formation at later time points. Taken together, *M. abscessus* is able to release bacteria from biofilms, and these bacteria can form secondary biofilms similar to those formed by the wild-type.

### Comparison between *M. abscessus* 19977 with other *M. abscessus* strains isolated from patients with cystic fibrosis

To verify whether the results obtained were only observed with the *M. abscessus* 19977 strain or were also seen with other strains isolated from patients with cystic fibrosis, the ability of the strain *M. abscessus* 00103 and *M. abscessus* 01715 were evaluated in comparison with the strain *M. abscessus* 19977 regarding the efficiency to form a new biofilm once it detaches from an existing biofilm. The results shown in **Table 2** suggest that all three strains behaved similarly and that detaching from a biofilm is a more efficient manner to establish a new biofilm than when bacteria were obtained from an agar plate.

**Table 2:** Ability of the strains of *M. abscessus* to form new biofilms after detachment.

Bacterial strains^1^	Biofilm mass^2^		
From plate	Day 2	Day 5	Day 7
19977	0.313 + 0.021	0.718 + 0.052	0.751 + 0.037
MAB 00103	0.202 + 0.047	0.722 + 0.039	0.756 + 0.026
MAB 01715	0.250 + 0.033	0.704 + 0.055	0.743 + 0.021
Satellite			
19977	0.323 + 0016	0.857 + 0.066	0.895 + 0.049
MAB 00103	0.303 + 0.029	0.894 + 0.034	0.884 + 0.037
MAB 01715	0.309 + 0.026	0.862 + 0.023	0.865 + 0.042

(1) *M. abscessus* strains 19977 (wildtype), 00103 (CF patient), and 01715 (CF patient).

(2) All statistical comparisons between strains and conditions tested were p > 0.05.

### Genes identified to play a role in bacterial release from biofilms

To better understand the mechanisms underpinning the release of *M. abscessus* from primary biofilms, an mmt7 transposon library was screened for mutant clones that do not release from primary biofilms in both HBSS and SCFM conditions. The mutants in this system were defined as being unable to establish a secondary biofilm. A supernatant containing released bacteria was utilized to form another biofilm, and each clone’s secondary biofilm was compared to that of the wild-type. In SCFM conditions, eight mutants were identified via ligation-mediated PCR (LM-PCR) and sequencing identified transposon insertion (**Table 3**). The transposon was inserted directly into two genes encoding hypothetical proteins [MAB_0274c (GXWXG domain), MAB_1222], although probable neighboring genes were affected if in an operon. Three membrane proteins [MAB_4706c (membrane-associated oxidoreductase complex, DoxX), MAB_2480 (Transmembrane), MAB_2301 (MmpL lipoprotein), MAB_0525c (LpqG lipoprotein)]. A chaperonin [MAB_0650 (GroEL)] and an enzyme [MAB_1277 (glycosyltransferase, modifies lipopolysaccharide]. In HBSS conditions, 15 mutants were selected and sequenced (**Table 4**). The transposon inserted into two tRNA’s (MAB_0275 and MAB_t5030c), along with two lipoproteins [MAB_3785c (LppF), MAB_0307c] and a translation elongation factor [MAB_1310 (BipA)]. Two genes encoded oxidoreductases [MAB_2438 (molybdopterin) and MAB_4628c]. One gene was a monooxygenase enzyme (MAB_4050c). A chaperonin [MAB_0650 (GroEL)] and a probable carbon starvation protein [MAB_1260c (CstA)] were also found. Two genes had domains suggesting kinase activity [MAB_3538 (DAGKc-domain) and MAB_1507 (hypothetical protein)]. An alkyl mercury lyase (MAB_4789c), a membrane protein [MAB_1170 (transports anions)], and transcriptional regulator (MAB_2089) were found. Further characterization is needed to understand the role these genes play in bacterial release from biofilms.

**Table 3:** Genes identified as deficient in releasing bacteria from SCFM biofilms.

Gene Disrupted	Probable Operon	Protein	Function	GC content (%)
MAB_4706c	MAB_4710c	Transcriptional regulator, TetR family	DNA binding	67.9
	MAB_4709c	Transcriptional regulator, AraC family	DNA binding/response to environmental stimuli	62.4
	MAB_4708c	Glyoxalase/bleomycin resistance protein	Metalloenzymes	62.8
	MAB_4707c	Lipase	Lipid catabolic process	65.6
	MAB_4706c	DoxX family protein	Membrane-associated oxidoreductase complex (MRC)	60.5
	MAB_4705c	Probable membrane protein, MmpS	Membrane protein, biosynthesis and secretion of siderophores	61.3
	MAB_4704c	Probable membrane protein, MmpL	MMPL family, lipid transport	58.6
	MAB_4703c	Probable membrane protein, MmpL	MMPL family, lipid transport	59.6
	MAB_4702c	Low molecular weight t-cell antigen tb8.4	Binds free heme in mycobacterial cytoplasm and then delivers to the membrane	61
MAB_2480	MAB_2480	Transmembrane protein		66.7
	MAB_2481	Integral membrane transporter	Major Facilitator Superfamily; Sugar (and other) transporter	65.6
	MAB_2482	Possible monooxygenase	Luciferase-like monooxygenase	63.4
between MAB_2301 MAB_2302	MAB_2300	Membrane protein, MmpS	Membrane protein, biosynthesis and secretion of siderophores	59.5
	MAB_2301	Membrane protein, mmpL	MMPL family, lipid transport	60.7
	MAB_2302	Probable conserved membrane protein, MmpS	Membrane protein, biosynthesis and secretion of siderophores	55.6
	MAB_2303	Membrane protein, MmpL	MMPL family, lipid transport	59.5
MAB_0274c	MAB_0274c	Conserved hypothetical protein	GXWXG protein	61.6
	MAB_0273c	Hypothetical Protein		64.3
MAB_0650	MAB_0650	60 kDa chaperonin 2 (Protein Cpn60 2) (GroEL)	TCP-1/cpn60 chaperonin family, ATP binding	66.3
MAB_0525c	MAB_0529c	Coenzyme Q (Ubiquinone) biosynthesis protein Coq4	Coenzyme Q (ubiquinone) biosynthetic pathway	62.8
	MAB_0528c	Probable epoxide hydrolase EphA	Catalytic activity	64.7
	MAB_0527c	Possible monooxygenase (Luciferase-like)	Flavin monooxygenase	62.6
	MAB_0526c	Hypothetical zinc-type alcohol dehydrogenase	Oxidoreductase activity	62.6
	MAB_0525c	Probable conserved lipoprotein LpqG	26 kDa periplasmic immunogenic proteins	66.7
	MAB_0524c	Probable conserved lipoprotein LpqG	26 kDa periplasmic immunogenic proteins	69
MAB_1277	MAB_1277	Glycosyltransferase RgtA/B/C/D-like domain	Modification of the lipopolysaccharide (LPS) inner core	66.2
	MAB_1278	O-acyltransferase WSD1-like N-terminal domain	Wax ester synthase-like Acyl-CoA acyltransferase domain	67.7
	MAB_1279	Probable 3-demethylubiquinone-9 3-methyltransferase		61.4
MAB_1222	MAB_1218	Probable aldehyde dehydrogenase AldA	Oxidizes a wide variety of aliphatic and aromatic aldehydes	65.1
	MAB_1219	Probable short-chain dehydrogenase/reductase	NAD- or NADP-dependent oxidoreductases	62.8
	MAB_1220	Probable dehydrogenase/reductase	Oxidoreductase activity	66.3
	MAB_1221	Possible 4-carboxymuconolactone decarboxylase	Degradation of aromatic compounds	58.7
	MAB_1222	Conserved hypothetical protein		62
	MAB_1223	Probable carboxylesterase (LipT)	Carboxylesterase family, active site serine	63.1
	MAB_1224	Conserved hypothetical protein		62.8
	MAB_1225	ABC transporter, permease protein	Transport of various osmoprotectants and nutrients	62
	MAB_1226	ABC transporter, ATP-binding protein	ABC transporter	61.4
	MAB_1227	ABC transporter, permease protein	Transport of various osmoprotectants and nutrients	67.3
	MAB_1228	ABC transporter, Glycine betaine	Transport system involved in bacterial osmoregulation	62.1
	MAB_1229	Transmembrane protein		66

**Table 4:** Genes identified as deficient in releasing bacteria from HBSS biofilms.

Gene Disrupted	Probable Operon	Protein	Function	GC content (%)
MAB_1260c	MAB_1260c	Probable carbon starvation protein (CstA)	Uptake and utilization of peptides, during carbon starvation	66
	MAB_1259c	Hypothetical Protein	Selenoprotein	65
	MAB_1258c	Hypothetical Protein (Transmembrane protein)		69
In between MAB_t5030c MAB_2089	MAB_2088	Transposase-like protein	DNA binding, transposition of insertion sequences	65.5
	MAB_2087	Transposase-like protein IS3/IS911	DNA binding	61.4
	MAB_t5030c	MAB_t5030c		63.8
	MAB_2089	Transcriptional regulator	DNA binding	65.5
	MAB_2090	Nucleotidyl transferase AbiEii/AbiGii toxin family protein	Type IV TA system, protects from phage infection	65.2
	MAB_2091	PNPLA domain-containing protein	Lipase and transacylase properties, roles in lipid and energy homeostasis	65
	MAB_2092	Nucleotidyltransferase	Second Messenger Oligonucleotide or Dinucleotide Synthetase domain	59.4
	MAB_2093	UBA/THIF-type NAD/FAD-binding fold	Ubiquitin E1-like enzymes that contain the NAD/FAD-binding fold	63.6
	MAB_2094	Hypothetical protein		58.4
	MAB_2095	Metal-dependent phosphohydrolase, HD subdomain	Phosphohydrolase activity	66.7
MAB_3785c	MAB_3786c	Uncharacterized protein		66
	MAB_3785c	Probable conserved lipoprotein LppF	Haloacid dehalogenase-like hydrolase	66.8
	MAB_3784c	Preprotein translocase secY subunit	Preprotein translocase pathway	62.2
	MAB_3783c	Adenylate kinase	Catalyzes the reversible transfer of MgATP to AMP	66.7
	MAB_3782c	Methionine aminopeptidase Map		66.9
MAB_4789c	MAB_4789c	Alkylmercury lyase	Detoxify mercurial compounds	62.3
	MAB_4788c	Mycothiol-dependent maleylpyruvate isomerase metal-binding	Metal-ion binding	65.2
	MAB_4787c	Hypothetical regulatory protein, TetR family	Controls the expression of MmpL lipid transporters	65.6
	MAB_4786c	Hypothetical protein (small)		63.3
MAB_2438	MAB_2438	Probable oxidoreductase	Molybdopterin oxidoreductase	64.6
	MAB_2439	Fluoride-specific ion channel FluC	Efflux transporter which confers resistance to fluoride ion	68.2
	MAB_2440	Fluoride-specific ion channel FluC	Efflux transporter which confers resistance to fluoride ion	63.2
	MAB_2441	UspA domain-containing protein	Provides a general “stress endurance” activity	62.4
MAB_1310	MAB_1310	Large ribosomal subunit assembly factor BipA	50S ribosomal subunit assembly protein	66.6
	MAB_1311	VOC domain-containing protein	Glyoxalase-like domain	64.8
	MAB_1312	Beta-lactamase-like	Alkyl sulfatase dimerization	63.1
	MAB_1313	Probable transcriptional regulator, AraC family	DNA binding	60
	MAB_1314	Mesocentin		67.2
	MAB_1315	Putative lipoprotein LpqW	Active transport of solutes	68.2
	MAB_1316	MshB	Mycothiol biosynthesis protein MshB	66.8
	MAB_1317	Integral membrane protein		64.7
MAB_0650	MAB_0650	60 kDa chaperonin 2 (Protein Cpn60 2) (GroEL)	TCP-1/cpn60 chaperonin family, ATP binding	66.3
MAB_3538	MAB_3538	DAGKc domain-containing protein	Kinase activity	67.3
	MAB_3539	Transcriptional regulator WhiB		67.1
MAB_4050c	MAB_4059c	Transcriptional regulator/sugar kinase	Transcriptional repressors, sugar kinases	67.8
	MAB_4058c	SRPBCC family protein	Polyketide synthesis	62.8
	MAB_4057c	D-inositol 3-phosphate glycosyltransferase	Glycosyltransferase of mycothiol biosynthesis	66.8
	MAB_4056c	YbjN domain-containing protein		62.7
	MAB_4055c	Acyl-CoA synthetase	AMP binding	64.6
	MAB_4054c	Pyridoxamine 5’-phosphate oxidase putative domain-containing protein	*de novo* biosynthesis of PLP	59.5
	MAB_4053c	Short chain dehydrogenase/reductase	NAD- or NADP-dependent oxidoreductases	67.3
	MAB_4052c	Lipase/esterase		67.5
	MAB_4051c	Reductase	Ferritin-like superfamily	60.3
	MAB_4050c	Probable monooxygenase	FAD, NAD binding	65.1
	MAB_4049c	2,3-bisphosphoglycerate-dependent phosphoglycerate mutase	Catalytic activity	64.2
	MAB_4048c	Sensor-like histidine kinase senX3	OmpR family	65.9
	MAB_4047c	Sensory transduction protein RegX3	OmpR family	64.5
	MAB_4046c	Putative transcriptional regulator, TetR family	DNA binding	63.4
	MAB_4045c	Probable ATP-binding protein ABC transporter	Biosynthesis of coenzyme Q	64
	MAB_4044c	Putative hydrolase/esterase/lipase		65.8
	MAB_4043c	Probable short-chain dehydrogenase/reductase	NAD- or NADP-dependent oxidoreductases	65.2
	MAB_4042c	Probable monooxygenase	Oxidoreductase activity	61.5
	MAB_4041c	Polyketide cyclase/dehydrase and lipid transport	Polyketide synthesis	64.2
	MAB_4040c	Conserved Hypothetical Protein		61.3
	MAB_4039c	Probable monooxygenase	Oxidoreductase activity	62.5
	MAB_4038c	Polyketide cyclase/dehydrase and lipid transport	Polyketide synthesis	65.9
MAB_0275	MAB_0275	Queuine tRNA-ribosyltransferase (TGT)		65.4
	MAB_0276	Probable cytochrome P450	Superfamily of heme-containing mono-oxygenases	63.1
MAB_0307c	MAB_0306c	Lipoprotein		64.2
	MAB_0307c	Lipoprotein		60.7
	MAB_0308c	Lipoprotein LpqN		63.2
MAB_1507	MAB_1507	Conserved hypothetical protein	Kinase activity	62.7
MAB_1170	MAB_1170	Probable membrane transporter protein	TauE, transport of anions across the cytoplasmic membrane	67.8
	MAB_1169	Hydrolase, alpha/beta fold		65.3
	MAB_1168	Exopolyphosphatase	Sugar kinase/actin/hsp70 superfamily	67.5
	MAB_1167	Septum formation initiator subfamily protein		67.9
	MAB_1166	Septum formation initiator		71.7
	MAB_1165	Enolase		66.9
	MAB_1164	Conserved lipoprotein LpqU	Lysozyme-like domain superfamily	68.1
MAB_4628c	MAB_4628c	Luciferase-like monooxygenase	Oxidoreductase activity	63.4

### Released *M. abscessus* are able to bind and invade epithelial cells

**Figure 3:**
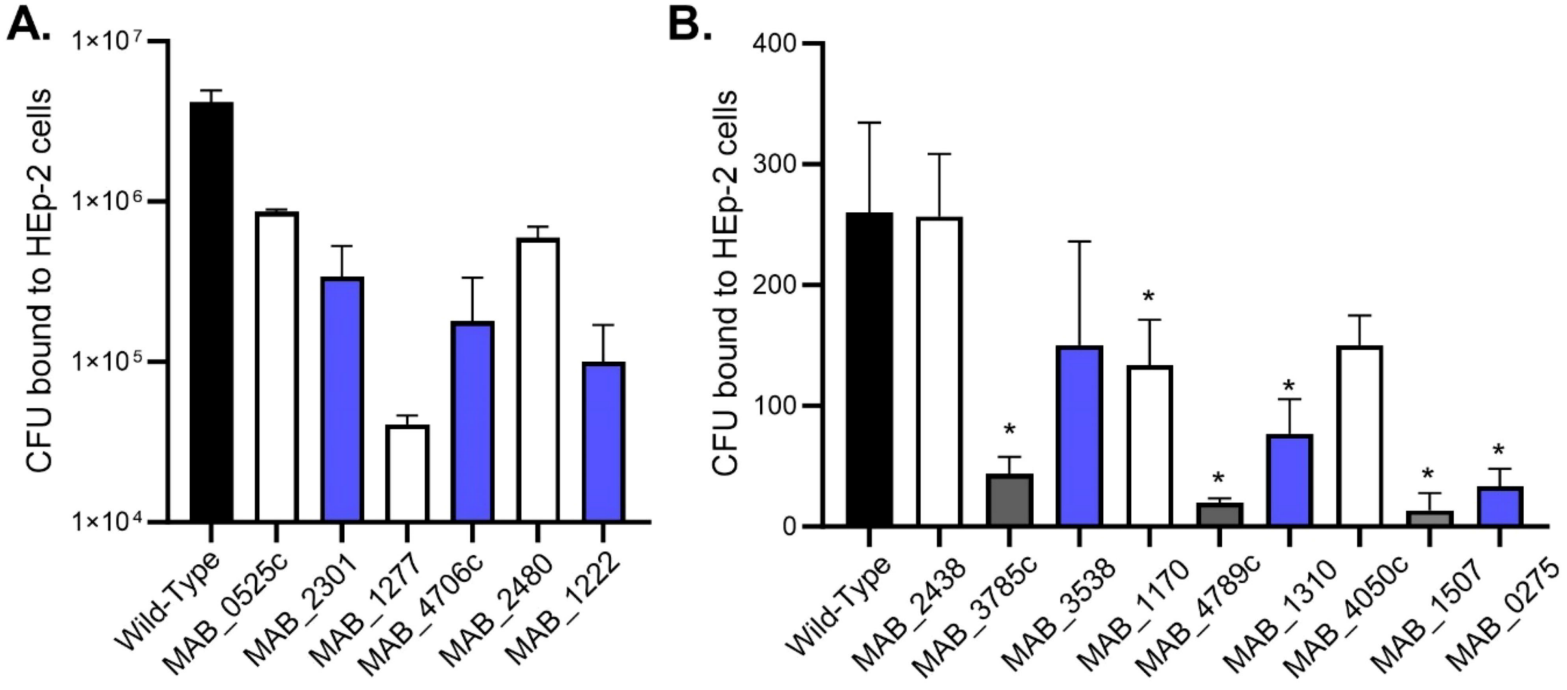
*M. abscessus secondary biofilm mutants were deficient in binding the host epithelium.* Wild-type and secondary biofilm deficient mutants identified in SCFM **(A)** or HBSS **(B)** media were incubated with HEp-2 epithelial cells at 4°C for 30 min. CFUs were recovered from epithelial cells. The error bars represent the SEM of 3 experiments. Statistical analysis was conducted using Brown–Forsythe and Welch ANOVA with a Dunnett’s T3 multiple comparison test. All mutants were compared to the wild-type; * indicates p < 0.05.

**Figure 4:**
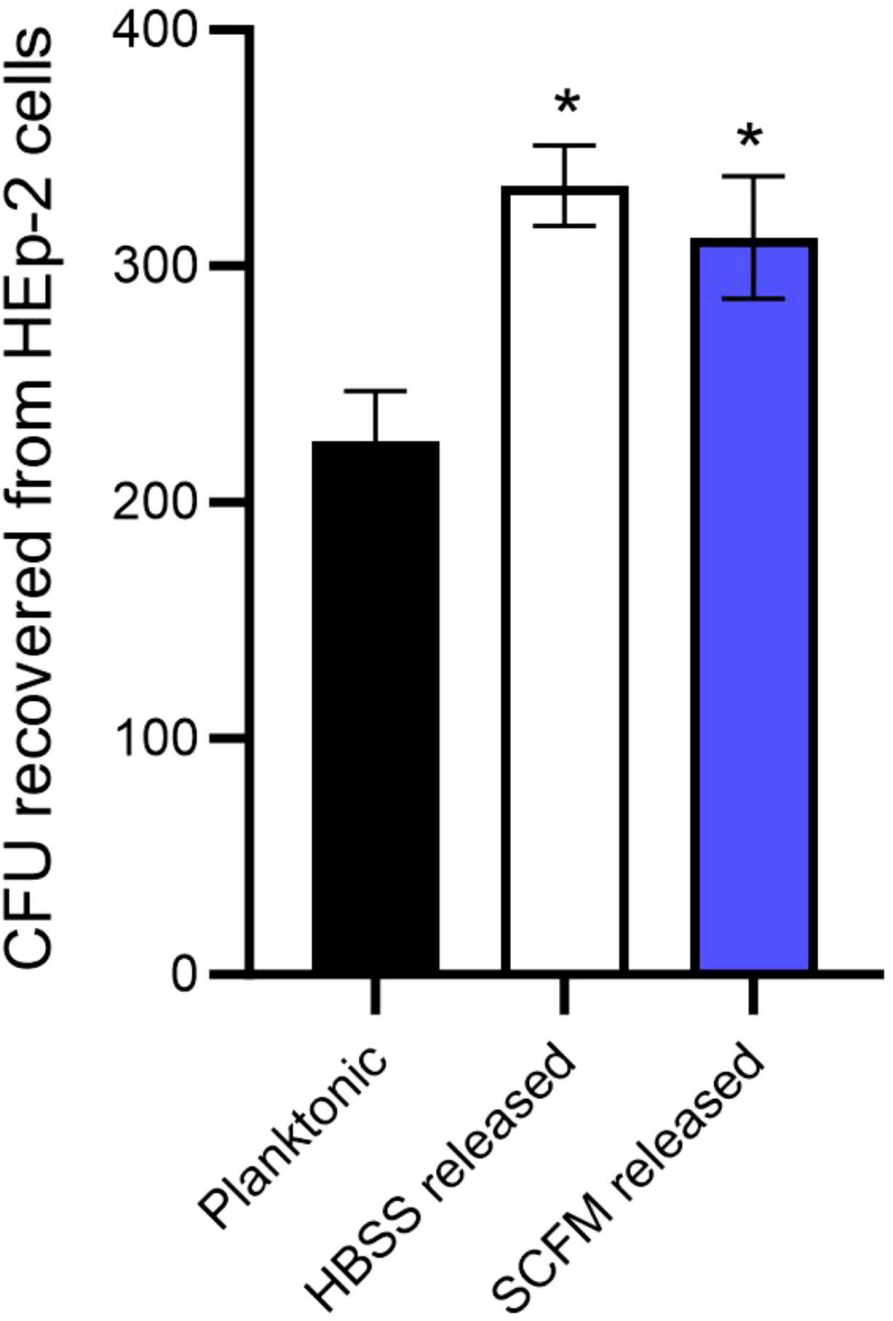
*M. abscessus bacteria released from biofilms are more efficient at invading the epithelial cells.* HEp-2 cells were infected with either planktonic *M. abscessus* or bacteria released from biofilms after 7 days in either HBSS or SCFM. The error bars represent SEM from three experiments. Statistical analysis was conducted using the Mann–Whitney t-test; * indicates p < 0.05.

*M. abscessus* lung infections are associated with biofilms, however, the infection likely originates with bacteria released from a biofilm in an environmental setting or from another patient. The bacteria encounter the lung epithelium and must be able to invade or establish a niche to form biofilms (12). The ability of *M. abscessus* to bind to respiratory epithelial cells was assayed by utilizing the wild-type *M. abscessus* 19977 and secondary biofilm mutants from the previous section (**Figure 3**). Unfortunately, no secondary biofilm mutants from SCFM demonstrated a significant reduction in binding epithelial capacity compared to the wild-type (**Figure 3A**). Six HBSS secondary biofilm mutants had significantly inhibited binding compared to wild-type *M. abscessus* 19977 (**Figure 3B**). Three of these binding deficient mutants are hypothetical proteins [MAB_1170 (membrane transporter for anions), MAB_4789c (Alky mercury lyase in an operon with TetR gene for regulating MmpL transporters), and MAB_1507 (conserved hypothetical protein with kinase activity)]. The observation of kinases and more transporter regulation are also key factors in the binding to epithelial cells and not just for the release from biofilms. Other mutants may encode enzymes or signals that are involved in the interaction with epithelial cells. Next, released bacteria from both the SCFM and HBSS biofilms were collected 7 days after formation and utilized to infect epithelial cells. The bacteria released from the biofilms, whether formed in HBSS or SCFM, significantly increased the invasion of epithelial cells compared to planktonic *M. abscessus* after 1 hour of infection (**Figure 4**). This finding further supports the hypothesis that the released bacteria phenotype from established biofilms is an adaptation suited not only for reforming biofilms but also establishing an infection in the lungs.

### Complementation of MAB_4706c restores the binding and secondary biofilm formation phenotype

**Figure 5:**
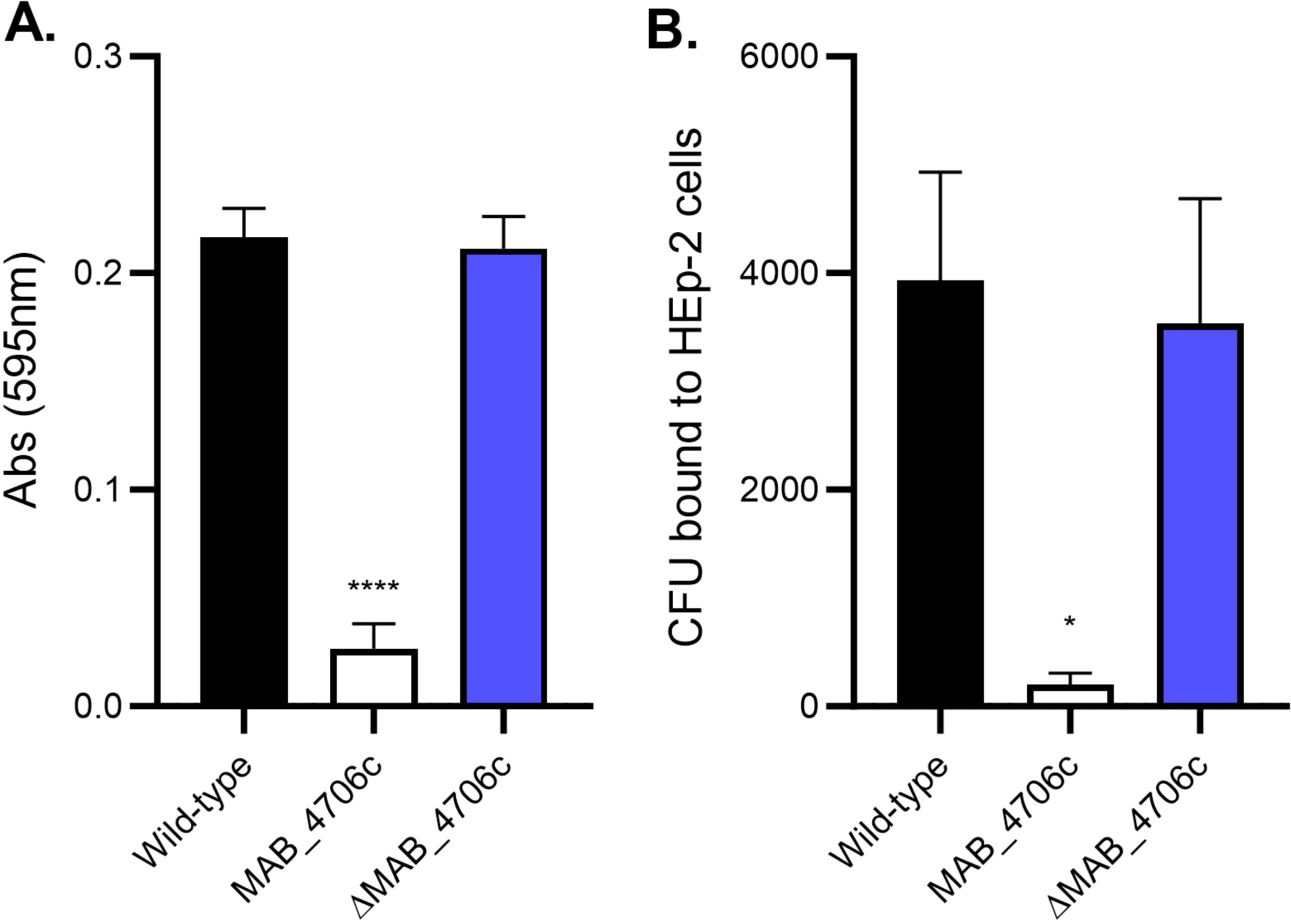
*Complementation of MAB_4706c restores the wild-type phenotype.* The ability of the bacteria to form secondary biofilms and bind to epithelial cells was determined. Wild-type *M. abscessus* 19977, mutant 4706c, and complemented MAB_4706c (ΔMAB_4706c) were utilized to form secondary biofilms **(A)** and assayed for epithelial binding **(B)**. Data are representative of three biological replicates. Statistical analysis was conducted using Brown–Forsythe and Welch ANOVA with a Dunnett’s T3 multiple comparison test; **** indicates p < 0.0001, * indicates p < 0.05.

MAB_4706c was cloned into pMV306 containing an apramycin-resistance gene and transformed into electrocompetent mutant MAB_4706c cells. Complemented MAB_4706c (ΔMAB_4706c) was tested against wild-type and mutant cells in its ability to form satellite biofilms and bind to epithelial cells (**Figure 5**). Secondary biofilms were formed in SCFM with wild-type, mutant 4706c, and ΔMAB_4706c, and absorbance readings were taken after 7 days (**Figure 5A**). ΔMAB_4706c had a similar optical density (O.D.) to wild-type *M. abscessus* 19977, while mutant 4706c biofilms had significantly decreased absorbance readings, suggesting complementation restores the bacteria’s ability to disperse from primary biofilms. The epithelial binding assay was repeated using the wild-type, mutant, and complemented bacteria (**Figure 5B**). ΔMAB_4706c had a similar binding capability to wild-type *M. abscessus* 19977, also restoring this phenotype. Taken together, the genes in the MAB_4706c operon play a major role in *M. abscessus* biofilm release and secondary attachment to the respiratory epithelium. MAB_4706c and MAB_4702c are both hypothetical proteins, while 4705c, 4704c, and 4703c are all membrane proteins in the MmpL family.

### Primary biofilm and secondary biofilm formation phenotypes of *M. abscessus* are able to invade macrophages and evade killing mechanisms

**Figure 6:**
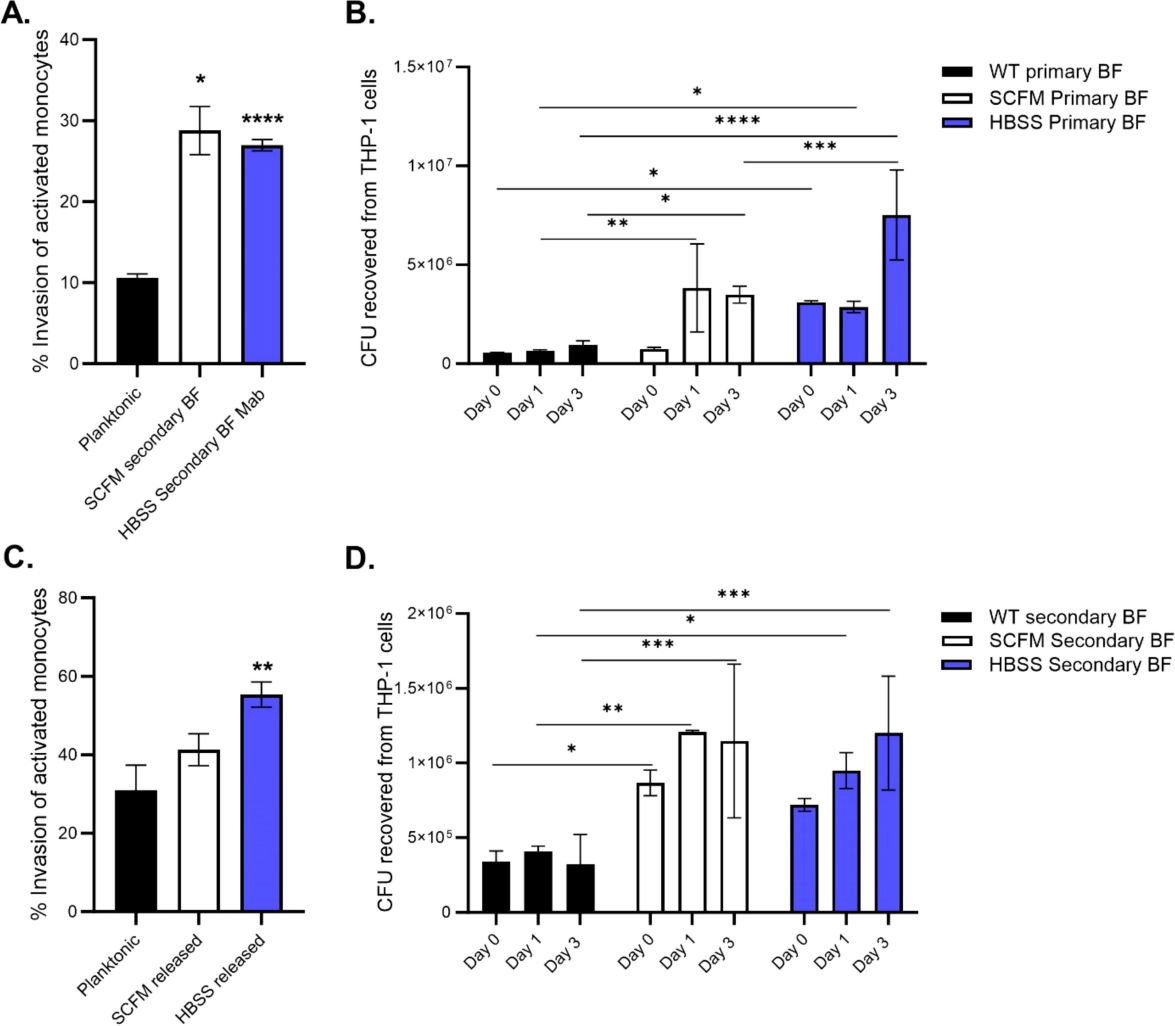
*Macrophages ingest M. abscessus biofilm phenotypes more efficiently than planktonic bacteria.* Bacteria from primary biofilms **(A, B)** or bacteria from secondary biofilms **(C, D)** were used to infect THP-1 macrophages. The invasion percentage **(A, C)** was determined after a 1-hour infection and surviving intracellular CFUs were obtained at days 0,1, and 3 **(B, D)** post-infection. Data are representative of two biological replicates. Statistical analysis was conducted using Brown–Forsythe and Welch ANOVA with a Dunnett’s T3 multiple comparison test **(A, C)** and 2-way ANOVA with Tukey’s multiple comparison test **(B, D)**; **** indicates p < 0.0001, *** indicates p < 0.001, ** indicates p < 0.01, * indicates p < 0.05.

Macrophages are the first immune cells that bacteria encounter in the lung environment, whether they are circulating airway macrophages or tissue macrophages after penetrating the epithelial layer. Mycobacteria are obligate intracellular pathogens and can disseminate the infection by infecting secondary macrophages in a transitionary manner. The ability of the bacteria collected from primary biofilms and those of released bacteria to form secondary biofilms means they are able to invade and replicate in macrophages (**Figure 6**). Disrupted primary biofilm bacteria were more efficient at invading macrophage monolayers compared to planktonic bacteria (**Figure 6A**). Both released bacteria and disrupted biofilm phenotypes were able to grow by day 3 within the macrophages (**Figures 6B, D**). Bacteria released from secondary biofilms had a similar uptake to plate bacteria but may not necessarily confer an advantage (**Figure 6C**). Overall, *M. abscessus* released from biofilms or in the biofilm phenotype were able to invade and grow intracellularly, regardless of whether the biofilms were made in SCFM or HBSS.

**Figure 7:**
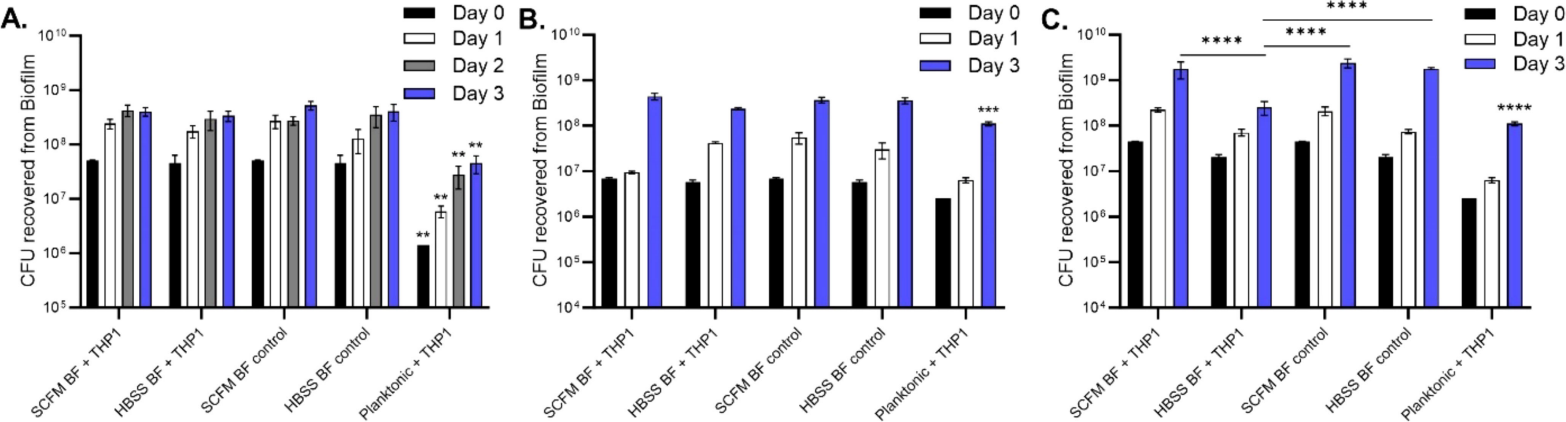
*Monocytes were unable to kill M. abscessus biofilms.* IFN-γ-stimulated monocytes were added to mature (7 day) biofilms formed in either SCFM or HBSS **(A)**. Monocytes were also added to replicative stage biofilms **(B)** and non-replicative stage biofilms **(C)**. The biofilms were disrupted and CFUs were determined at days 1, 2, or 3 post cell addition for biofilm survival. Planktonic bacteria served as a macrophage killing control. Data are representative of three biological replicates. Statistical analysis was conducted using 2-way ANOVA with Tukey’s multiple comparison test. **** indicates p < 0.0001, *** indicates p < 0.001, ** indicates p < 0.01.

Next, IFN-γ-stimulated monocytes were added to mature biofilms in both SCFM and HBSS to determine whether the cells could eliminate biofilm CFUs (**Figure 7**). Monocytes did not decrease any of the biofilm CFUs at all time points: 24, 48, and 72 hours post addition (**Figure 7A**). Planktonic bacteria were significantly reduced by monocytes compared to the bacteria protected by biofilms. THP-1 monocytes were also added during the biofilm formation process in replicative stage biofilm (day 2) and non-replicative stage biofilm (day 5) (**Figures 7B, C**, respectively). The addition of phagocytic cells to biofilms did not contribute to a decrease in biofilm CFUs during the replication stage. However, during the stationary phase, biofilms established in HBSS were significantly reduced by day 3 compared to the HBSS or SCFM biofilm control. As before, the planktonic bacteria control had a significant reduction in CFUs compared to its biofilm counterparts. Taken together, bacteria released from biofilms or in the biofilm phenotype are able to combat macrophage killing but only confer an advantage if formed in SCFM and during the non-replicative stage of formation.

### Bacterial surface proteins

**Figure 8:**
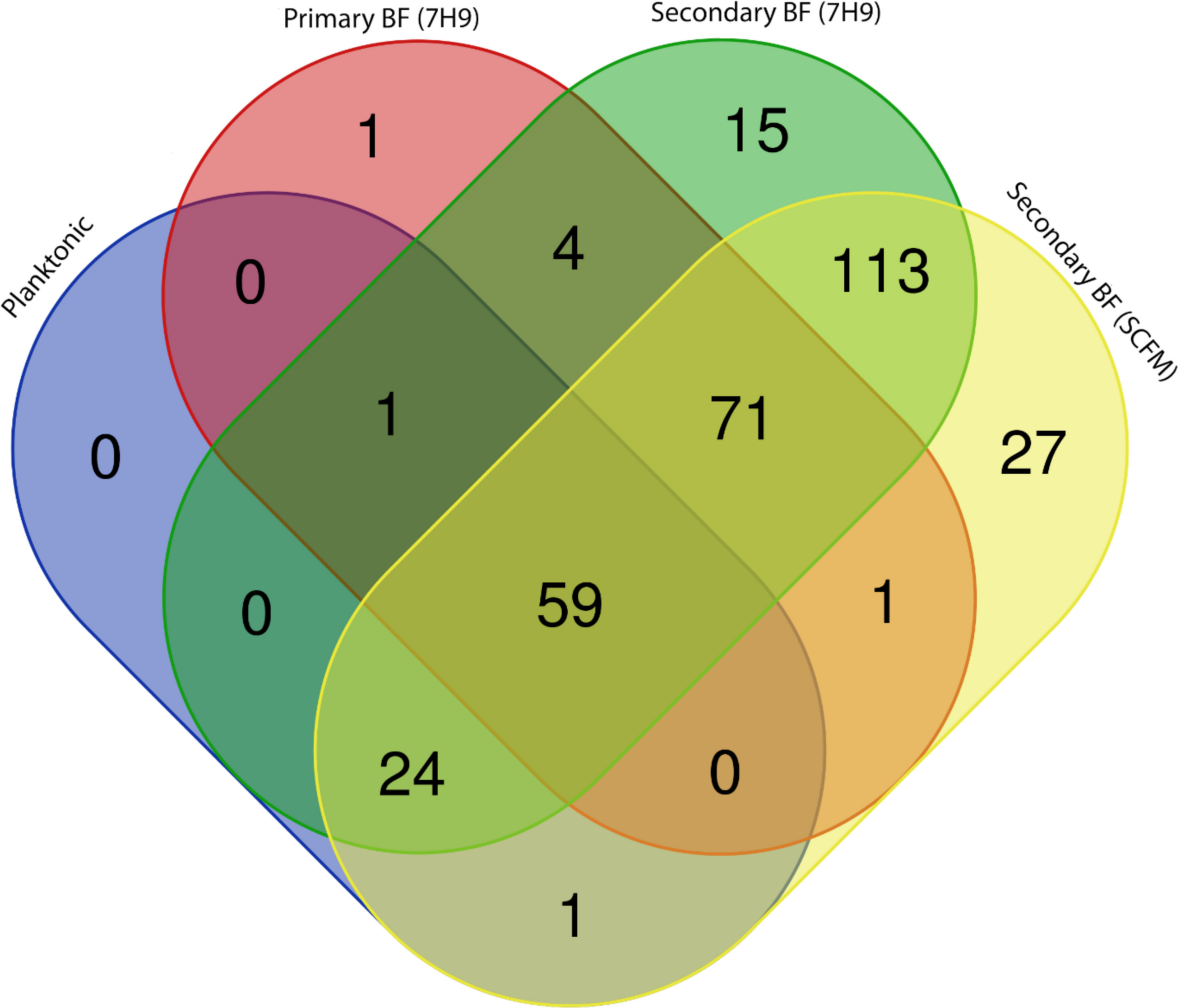
A *Venn diagram depicting proteins determined through proteomic analysis.* Each treatment group’s list of proteins was compared to each other to resolve overlaps. Graphical representation of **Tables 1**, **5**–**14**. https://bioinformatics.psb.ugent.be/webtools/Venn/.

The release of bacteria from biofilms may express a set of different proteins on the surface that then make them more capable of seeding and infecting different sites or hosts. We compared planktonic bacteria grown in 7H10 agar, bacteria with the biofilm phenotype (7 days biofilm), bacteria released from biofilm when in Middlebrook 7H9 broth, and bacteria released from biofilm in SCFM. The results, shown in a Venn diagram, demonstrated that bacteria released from biofilm expressed significantly more proteins than bacteria grown in plates (**Figure 8**). The bacteria found in biofilms accounted for 295 of the 380 proteins detected and are summarized in **Tables 5**–**15**. Planktonic bacteria accounted for 85 of the 380 proteins and are summarized in **Tables 5**, **6**, **8**, **15** and all the listed proteins overlapped with the other treatment groups examined. The bacteria released from biofilms expressed more proteins on their surface, suggesting a remarkable change in phenotype. It is notable that many of the proteins were different lipoproteins, of a small size, and uncharacterized, and there were many types of enzymes. We compared the transposon mutants that were found deficient in secondary biofilm formation to the surface proteins detected. Three mutants, MAB_0525c (all treatments), MAB_2301 [planktonic, secondary BF (7H9), secondary BF (SCFM), and primary BF (7H9)], and MAB_3538 [secondary BF (7H9) and secondary BF (SCFM)] were found in the proteomic lists. Interestingly, the latter mutant, MAB_3538, with kinase activity, was only found in the released biofilm groupings.

**Table 5:** Surface proteins unique to planktonic bacteria, primary biofilm (BF) (7H9), and secondary BF (7H9).

	Accession	Description	# AAs	MW [kDa]	Ensembl Gene ID
1	B1MFZ3	NLPC_P60 domain-containing protein	450	44.3	MAB_3664

**Table 6:** Surface proteins identified in planktonic bacteria, secondary biofilm (BF) (7H9), and secondary BF (SCFM).

	Accession	Description	# AAs	MW [kDa]	Ensembl Gene ID
1	B1MNX2	Uncharacterized protein	249	26.9	MAB_1940c
2	B1MKD0	Probable membrane protein, MmpL	981	107.3	MAB_1134c
3	B1MAN4	Conserved hypothetical membrane protein	317	33.6	MAB_2224c
4	B1MAW1	Putative membrane protein, mmpL	984	107.9	MAB_2301
5	B1MB89	Probable NADH dehydrogenase (NDH)	484	52	MAB_2429c
6	B1MCR7	Uncharacterized protein	682	73.5	MAB_2960
7	B1MLX5	Possible lipoprotein peptidase LpqM	484	52.4	MAB_1466c
8	B1MB39	Hypothetical lipoprotein LpqH	162	15.6	MAB_2379
9	B1MGH4	Uncharacterized protein (SHOCT domain-containing protein)	266	28.8	MAB_3845c
10	B1MMD6	Probable cation-transporting ATPase G	653	66.6	MAB_4853c
11	B1MJ98	Probable cation-transporting ATPase E	800	84.1	MAB_0962
12	B1MNZ9	Cytochrome bc1 complex cytochrome c subunit	295	31.1	MAB_1968c
13	B1MMM0	Uncharacterized protein	1144	118.4	MAB_4937
14	B1MDL5	Uncharacterized protein	245	26.9	MAB_3258c
15	B1MG09	Strictosidine synthase family protein	342	35.9	MAB_3680
16	B1MFL7	MurNAc-LAA domain-containing protein	272	27.8	MAB_0318c
17	B1MKQ2	Uncharacterized protein (lipid droplet-associated protein)	231	25.1	MAB_1256c
18	B1MEJ2	Iron-sulfur cluster-binding protein, RIESKE family	517	58.1	MAB_0156c
19	B1MC44	Probable macrolide ABC transporter, ATP-binding protein	542	58.6	MAB_2736c
20	B1MKK5	Isoprenyl transferase	260	29.5	MAB_1209
21	B1MBM0	Uncharacterized protein (LGFP repeat)	201	20.5	MAB_2560
22	B1MI34	Uncharacterized protein (CAS/CSE protein)	306	32.7	MAB_0760c
23	B1MAY5	Putative short-chain dehydrogenase/reductase	318	33.8	MAB_2325
24	B1MFL0	FAD-binding PCMH-type domain-containing protein	456	51.2	MAB_0311c

**Table 7:** Surface proteins unique to primary biofilm (BF) (7H9), secondary BF (7H9), and secondary BF (SCFM).

	Accession	Description	# AAs	MW [kDa]	Ensembl Gene ID
1	B1MJ65	Uncharacterized protein (p-aminobenzoate N-oxygenase AurF)	351	40.3	MAB_0929
2	B1MKG2	Uncharacterized protein (Septum formation initiator)	213	22.4	MAB_1166
3	B1MAK0	DUF4333 domain-containing protein	271	28.7	MAB_2190
4	B1MJ23	Hypothetical lipoprotein lpqH	158	15.5	MAB_0885c
5	B1MAN6	Conserved hypothetical transmembrane protein	462	47	MAB_2226c
6	B1MIZ5	Putative Fe-S oxidoreductase	976	103.7	MAB_4293
7	B1MIG7	Putative membrane protein, MmpL	987	107.6	MAB_4115c
8	B1MIS4	Uncharacterized protein	442	47.3	MAB_4222
9	B1MK70	Putative membrane protein, MmpL	1001	107.6	MAB_4508
10	B1MIW6	Uncharacterized protein (secreted)	243	26.9	MAB_4264c
11	B1MB68	Uncharacterized protein (von Willebrand factor, type A)	611	62.9	MAB_2408c
12	B1MMP0	Uncharacterized protein	78	8.4	MAB_1516
13	B1MII0	Uncharacterized protein	263	27.8	MAB_4128c
14	B1ME75	Possible serine/threonine phosphatase Ppp	501	52.3	MAB_0037c
15	B1MIG8	Putative membrane protein, MmpL	959	104.1	MAB_4116c
16	B1MGJ6	Uncharacterized protein (Inhibition of morphological differentiation protein)	293	31.2	MAB_0431c
17	B1MHK5	Uncharacterized protein	418	44.5	MAB_4012c
18	B1MAP0	Hypothetical PPE family protein	523	53.9	MAB_2230c
19	B1MAW3	Putative membrane protein, MmpL	997	109.7	MAB_2303
20	B1MB23	Uncharacterized protein (Channel-forming protein)	310	31.9	MAB_2363
21	B1MB79	PknH_C domain-containing protein	269	28.6	MAB_2419c
22	B1ME72	Putative serine/threonine-protein kinase PknA	420	44.2	MAB_0034c
23	B1MPH0	NADH-quinone oxidoreductase	792	84	MAB_2140
24	B1MFV7	Bifunctional membrane-associated penicillin-binding protein PonA2/glycosyl transferase	811	85.2	MAB_0408c
25	B1MGB9	Possible protease IV SppA (Endopeptidase IV)	583	61.6	MAB_3789c
26	B1MIZ3	Uncharacterized protein (Proline-rich protein)	605	62.8	MAB_4291
27	B1MMP8	Uncharacterized protein (CBS domain-containing protein)	429	46.8	MAB_1524c
28	B1MIQ8	Putative amino acid transporter	513	54.6	MAB_4206
29	B1ML01	NAD(P) transhydrogenase subunit beta	481	49.7	MAB_4577c
30	B1MEW8	UPF0182 protein MAB_3498c	988	107.1	MAB_3498c
31	B1MNZ8	Probable ubiquinol-cytochrome c reductase iron-sulfur subunit (Rieske iron-sulfur protein)	391	42.9	MAB_1967c
32	B1MF58	Lipoprotein LpqB	584	61.7	MAB_3589c
33	B1MEU5	Cell division ATP-binding protein FtsE	229	25.6	MAB_3475c
34	B1MEM5	Probable arabinosyltransferase C	1085	116.7	MAB_0189c
35	B1MPH3	NADH-quinone oxidoreductase subunit J	259	27.1	MAB_2143
36	B1MGU8	ATP-dependent zinc metalloprotease FtsH	750	80.5	MAB_0533
37	B1MJ91	Probable sensor histidine kinase PrrB	470	50.6	MAB_0955c
38	B1MCV2	Trk system potassium uptake protein CeoC	219	23.8	MAB_2995
39	B1MCI8	Protein translocase subunit SecD	586	61.4	MAB_2880c
40	B1MB57	Protein translocase subunit SecA	772	84.8	MAB_2397
41	B1MLG6	Probable short-chain dehydrogenase/reductase	268	28.9	MAB_1305
42	B1MPE7	Hypothetical short-chain dehydrogenase/reductase	293	31	MAB_2117
43	B1MHE0	Possible oxidoreductase	295	31.9	MAB_3947
44	B1MDZ7	Probable FeIII-dicitrate-binding periplasmic lipoprotein	346	36.2	MAB_3390
45	B1ML03	Probable NAD(P) transhydrogenase, alpha1 subunit PntAA	362	37.4	MAB_4579c
46	B1MJQ0	Putative short-chain dehydrogenase/reductase	283	29.6	MAB_4335
47	B1ME27	Putative short-chain dehydrogenase/reductase	294	31.9	MAB_3420c
48	B1MJH3	Putative short-chain dehydrogenase/reductase	249	26.2	MAB_1039
49	B1MEK1	Putative acyltransferase	268	29.1	MAB_0165
50	B1MCE3	Uncharacterized protein (Deazaflavin-dependent nitroreductase)	142	15.2	MAB_2835
51	B1MHK2	Uncharacterized protein (Deazaflavin-dependent nitroreductase)	151	16.8	MAB_4009c
52	B1MJQ4	Saccharopine_dehydrogenase_NADP domain-containing protein	415	44.3	MAB_4339c
53	B1MH29	Putative acyl-CoA dehydrogenase	387	42.8	MAB_0615
54	B1MJI2	Probable class II aldolase	268	29.3	MAB_1048c
55	B1MIL0	Probable acyl-CoA dehydrogenase FadE	392	43.1	MAB_4158
56	B1MH42	AAA_31 domain-containing protein	1041	109.9	MAB_0628
57	B1MJY6	Putative succinate dehydrogenase, iron-sulfur subunit	248	28.4	MAB_4423
58	B1MNU9	Diacylglycerol O-acyltransferase	457	49.5	MAB_1917
59	B1MNU7	Probable fatty-acid-coa ligase FadD	600	65	MAB_1915
60	B1MB08	Possible long-chain acyl-CoA synthase	989	105.5	MAB_2348
61	B1MKW1	Uncharacterized protein (L,D-ATPase catalytic domain-containing protein)	333	35.7	MAB_4537c
62	B1MLH3	Beta-lactamase-like	633	69.4	MAB_1312
63	B1MEL5	Probable fatty-acid-CoA ligase FadD	637	68.9	MAB_0179
64	B1MGS2	Diacylglycerol O-acyltransferase	461	50.3	MAB_0507
65	B1MFZ9	Adenosine deaminase	362	39.4	MAB_3670c
66	B1MHH4	Probable zinc metalloprotease	691	75.8	MAB_3981c
67	B1MEL2	Antigen 85-A	333	35.8	MAB_0176
68	B1MEL3	Antigen 85-A/B/C	325	34.9	MAB_0177
69	B1MB96	Uncharacterized protein (Alanine and proline-rich secreted protein Apa)	344	34.8	MAB_2436
70	B1MEL1	Antigen 85-C	324	34.7	MAB_0175
71	B1MDH6	Chromosomal replication initiator protein DnaA	491	55	MAB_0001

**Table 8:** Unique proteins identified in planktonic bacteria and secondary biofilm (SCFM).

	Accession	Description	# AAs	MW [kDa]	Ensembl Gene ID
1	B1MLS5	Putative lipoprotein LprB	195	20.6	MAB_1416

**Table 9:** Surface proteins unique to primary biofilm (BF) (7H9) and secondary BF (7H9).

	Accession	Description	# AAs	MW [kDa]	Ensembl Gene ID
1	B1MID7	Uncharacterized protein	447	49.5	MAB_4085c
2	B1MJV4	Putative ABC transporter, periplasmic substrate-binding	330	34.7	MAB_4390
3	B1MK78	Hypothetical porin (MspA)	223	23.4	MAB_1080
4	B1MJB0	Uncharacterized protein (PE-PGRS family protein)	606	61.2	MAB_0974

**Table 10:** Surface proteins unique to primary biofilm (BF) (7H9) and secondary BF (SCFM).

	Accession	Description	# AAs	MW [kDa]	Ensembl Gene ID
1	B1MAX9	Lysylphosphatidylglycerol biosynthesis bifunctional protein LysX	1110	122.1	MAB_2319c

**Table 11:** Surface proteins unique to secondary biofilm (BF) (7H9) and secondary BF (SCFM).

	Accession	Description	# AAs	MW [kDa]	Ensembl Gene ID
1	B1MM28	Putative membrane protein, MmpS	147	15.2	MAB_4745
2	B1MGS5	Probable non-ribosomal peptide synthase	1342	141.6	MAB_0510c
3	B1MKJ2	Proline-rich antigen (36 kDa antigen)	190	19.5	MAB_1196
4	B1MEE6	Conserved hypothetical membrane protein	222	23.3	MAB_0110c
5	B1MDT9	Uncharacterized protein (DoxX family protein)	268	28.4	MAB_3332c
6	B1MC32	VWFA domain-containing protein	336	35.9	MAB_2724c
7	B1MFX2	Conserved hypothetical membrane protein	173	18.7	MAB_0423c
8	B1MEM8	Probable oxidoreductase	507	55.1	MAB_0192c
9	B1MHI8	Uncharacterized protein (hydrolase)	305	32.6	MAB_3995
10	B1MEF0	Uncharacterized protein	371	40	MAB_0114
11	B1MKI6	Uncharacterized protein	281	29	MAB_1190
12	B1MJG4	Uncharacterized protein	136	15.7	MAB_1030
13	B1MP25	Probable conserved lipoprotein LppM	226	24.1	MAB_1994c
14	B1MK66	Uncharacterized protein	481	52.4	MAB_4504c
15	B1MHY2	Uncharacterized protein	600	65.4	MAB_0708
16	B1MGV4	Conserved hypothetical transmembrane protein	493	53.7	MAB_0539
17	B1MBP9	Uncharacterized protein	257	28.9	MAB_2589c
18	B1MHR9	Uncharacterized protein	313	32.4	MAB_4077
19	B1ML18	Putative Mce family protein	452	47.6	MAB_4594c
20	B1MB71	Uncharacterized protein (CBS domain-containing protein)	453	48.1	MAB_2411c
21	B1MJY4	Putative succinate dehydrogenase	269	30.7	MAB_4421
22	B1MG85	Putative FtsK/SpoIIIE family protein	1311	142.3	MAB_3756c
23	B1MJD8	Uncharacterized protein	229	24.6	MAB_1004c
24	B1ML23	Putative Mce family protein	510	52.6	MAB_4599c
25	B1MM24	Putative terminal quinol oxidase, subunit I	341	37	MAB_4741c
26	B1MP49	Uncharacterized protein	154	15.8	MAB_2018
27	B1ML20	Putative Mce family protein	379	40.2	MAB_4596c
28	B1MCI2	Probable peptidyl-prolyl cis-trans isomerase	307	32.3	MAB_2874
29	B1MEK9	UbiA prenyltransferase family protein	304	32.8	MAB_0173
30	B1MKU5	Uncharacterized protein (AI-2E family transporter)	399	41.8	MAB_4521c
31	B1ME28	Uncharacterized protein	291	31	MAB_3421
32	B1MIY5	Uncharacterized protein (Dynamin N-terminal domain-containing protein)	627	68.5	MAB_4283c
33	B1MNZ5	Uncharacterized protein	240	24.2	MAB_1964
34	B1ME59	DUF3566 domain-containing protein	313	33	MAB_0020
35	B1MJI6	Uncharacterized protein	272	28.4	MAB_1052c
36	B1MB80	PknH_C domain-containing protein	251	26.5	MAB_2420c
37	B1MNZ7	Probable ubiquinol-cytochrome c reductase cytochrome b subunit	546	60.4	MAB_1966c
38	B1MPG9	NADH-quinone oxidoreductase subunit F	434	46.7	MAB_2139
39	B1MAF6	NADH-quinone oxidoreductase, M subunit NuoM	535	56.8	MAB_2146
40	B1MK76	Putative serine protease	437	43.4	MAB_1078
41	B1MFX0	Possible membrane-associated serine protease	421	43.3	MAB_0421
42	B1MH83	Probable histidine kinase response regulator	567	60.1	MAB_3890c
43	B1MGH1	Probable cationic amino acid transport integral membrane protein	491	51.3	MAB_3842
44	B1MM45	DUF3533 domain-containing protein	648	68.5	MAB_4762
45	B1MEN9	Probable o-antigen/lipopolysaccharide transport ATP-binding protein ABC transporter RfbE	263	28.7	MAB_0203c
46	B1MAS1	Hypothetical ABC transporter ATP-binding protein	582	61.7	MAB_2261c
47	B1MF69	Putative cation transporter	307	32.4	MAB_3600c
48	B1MN95	Membrane protein OxaA	361	40.4	MAB_4953c
49	B1MPG7	NADH-quinone oxidoreductase subunit D	432	47.6	MAB_2137
50	B1ML95	Probable ABC transporter, permease protein	368	38.7	MAB_4672c
51	B1MP37	UDP-N-acetylglucosamine–N-acetylmuramyl-(pentapeptide) pyrophosphoryl-undecaprenol N-acetylglucosamine transferase	382	40.1	MAB_2006
52	B1MEU4	Cell division protein FtsX	300	32.8	MAB_3474c
53	B1MEM2	Probable arabinosyltransferase A	1097	116.5	MAB_0186c
54	B1MEH0	Probable pyruvate dehydrogenase	579	62.1	MAB_0134c
55	B1MJH6	Cytochrome c oxidase subunit 1	520	57.7	MAB_1042c
56	B1MLV8	ATP synthase subunit b	177	18.5	MAB_1449
57	B1MPG6	NADH-quinone oxidoreductase subunit C	228	25.6	MAB_2136
58	B1MDZ6	Cytochrome c oxidase subunit 1	564	62.6	MAB_3389c
59	B1MAN5	Probable peptidase	461	46.5	MAB_2225c
60	B1MF59	Probable sensor histidine kinase MtrB	576	62.6	MAB_3590c
61	B1MNV9	Putative sensor-type histidine kinase PrrB	429	45.8	MAB_1927
62	B1MEU7	MscS Mechanosensitive ion channel	350	38	MAB_3477c
63	B1MIU8	Putative Na+/H+ antiporter	555	59.1	MAB_4246
64	B1MGL4	Probable copper-transporting ATPase	646	66.9	MAB_0449
65	B1MLN4	Probable sugar ABC transporter, ATP-binding protein SugC	391	42	MAB_1375
66	B1MFQ4	Ammonium transporter	441	45.6	MAB_0355
67	B1MAS2	Hypothetical ABC transporter ATP-binding protein	862	93.9	MAB_2262c
68	B1MKF5	Putative iron permease FTR1	683	71.6	MAB_1159
69	B1MCU8	Na(+)/H(+) antiporter NhaA	441	46.2	MAB_2991c
70	B1MGB4	Protein translocase subunit SecY	419	45.6	MAB_3784c
71	B1MCI7	Protein-export membrane protein SecF	424	45	MAB_2879c
72	B1MPD4	Dihydroorotate dehydrogenase (quinone)	353	37.2	MAB_2104c
73	B1MHZ3	Putative oligopeptide ABC transporter,solute-binding protein	528	58	MAB_0719
74	B1MFX5	Probable peptide ABC transporter DppA	539	59.7	MAB_0426
75	B1MC01	Uncharacterized protein (nitroreductase)	145	16.1	MAB_2692c
76	B1MEK2	Putative acyltransferase	254	27.7	MAB_0166
77	B1ML94	Probable ABC transporter, ATP-binding protein	238	25.1	MAB_4671c
78	B1MJE4	Putative MCE family protein	498	52.1	MAB_1010c
79	B1MN49	Isoprenyl transferase	302	34.6	MAB_1676
80	B1MDD7	Probable lipoprotein LppI	235	24.1	MAB_3181
81	B1MLK3	Probable fatty-acid-CoA ligase FadD	596	65	MAB_1342
82	B1MF08	DAGKc domain-containing protein	325	34.7	MAB_3538
83	B1MH44	CbiA domain-containing protein	638	67.9	MAB_0630
84	B1MCU4	Uncharacterized protein (Outer membrane receptor protein)	175	18.7	MAB_2987
85	B1MNT8	Pyridoxamine 5’-phosphate oxidase-related	267	28.2	MAB_1906
86	B1MD78	Putative acyl-CoA dehydrogenase	413	44.6	MAB_3122
87	B1MGZ0	Putative ABC transporter ATP-binding protein	261	28.3	MAB_0576c
88	B1MGW2	Putative iron compound ABC transporter	330	35.8	MAB_0547c
89	B1MGE0	Putative hydrolase, alpha/beta fold	286	31.5	MAB_3810
90	B1MGD1	Uncharacterized protein (VCBS repeat-containing protein)	179	18.5	MAB_3801c
91	B1MPG8	NADH-quinone oxidoreductase, E subunit NuoE	247	26.7	MAB_2138
92	B1MBT5	Putative ABC transporter/extracellular ligand-binding receptor	394	40.9	MAB_2626c
93	B1MGY1	Putative lipoprotein lpqE	227	23.2	MAB_0567c
94	B1MH09	Probable acyl-CoA dehydrogenase FadE	386	42.5	MAB_0595c
95	B1MGC0	Uncharacterized protein (Sensor domain-containing protein)	527	56.9	MAB_3790
96	B1MGM8	Probable oxidase (Copper-binding protein)	512	54.1	MAB_0463c
97	B1MHH6	Uncharacterized protein (Lipoprotein)	194	20.4	MAB_3983c
98	B1MH57	Putative short-chain dehydrogenase/reductase	304	32.2	MAB_0643
99	B1MMK7	Uncharacterized protein (proline rich 28 kDa antigen)	321	32	MAB_4924
100	B1MEX3	Putative ABC transporter, ATP-binding protein	474	52.3	MAB_3503
101	B1MM44	Uncharacterized protein (ABC transporter)	241	26	MAB_4761
102	B1MP15	Probable 1-acylglycerol-3-phosphate O-acyltransferase	245	26.7	MAB_1984
103	B1MLZ8	Probable acyl-CoA ligase FadD	598	64.2	MAB_1489
104	B1MJ71	Putative fatty-acid–CoA ligase FadD	572	61.9	MAB_0935c
105	B1MDX4	Putative fatty-acid-CoA ligase	1163	127.5	MAB_3367
106	B1MPF0	Putative polyketide synthase MbtC	441	45.4	MAB_2120c
107	B1MCR9	Probable fatty-acid-CoA ligase FadD	1184	127.8	MAB_2962
108	B1MB93	Molybdenum ABC transporter ModA, periplasmic	273	27.4	MAB_2433
109	B1MG92	Cutinase	228	23.5	MAB_3763
110	B1MNN1	Neutral metalloproteinase	347	37.2	MAB_1849
111	B1MFL1	Enolase	424	44.1	MAB_0312c
112	B1MN45	GTPase Era	304	33.4	MAB_1672
113	B1MEI3	Uncharacterized protein (ESX secretion-associated protein EspG)	247	26.5	MAB_0147c

**Table 12:** Surface proteins unique to primary biofilm (7H9).

	Accession	Description	# AAs	MW [kDa]	Ensembl Gene ID
1	B1MN43	Uncharacterized protein (VBS domain-containing protein)	435	46.9	MAB_1670

**Table 13:** Surface proteins unique to secondary biofilm (7H9).

	Accession	Description	# AAs	MW [kDa]	Ensembl Gene ID
1	B1MP27	Conserved hypothetical transmembrane protein	134	14.3	MAB_1996
2	B1MJD9	Putative MCE family protein	416	44.3	MAB_1005c
3	B1MET1	Uncharacterized protein (Nitroreductase)	142	16.1	MAB_3461c
4	B1MCG8	Uncharacterized protein (Deazaflavin-dependent nitroreductase)	148	16.3	MAB_2860c
5	B1MF62	Possible Mg2+ transport P-type ATPase C MgtC	240	25.4	MAB_3593
6	B1MPH1	NADH-quinone oxidoreductase subunit H	422	45.3	MAB_2141
7	B1MJK9	50S ribosomal protein L32	57	6.6	MAB_1075
8	B1MJL2	Uncharacterized protein	554	55.4	MAB_4296c
9	B1MFZ2	Uncharacterized protein (protein secretion)	108	10.7	MAB_3663
10	B1MGX7	4HBT domain-containing protein	182	19.2	MAB_0563
11	B1ME15	Uncharacterized protein (Maleypyruvate isomerase family mycothiol-dependent enzyme, MDMPI)	245	26.7	MAB_3408c
12	B1MDU0	Probable conserved lipoprotein LppZ	387	39.9	MAB_3333
13	B1MBY8	Uncharacterized protein (PE-PGRS family protein)	576	57.9	MAB_2679
14	B1MPE9	Putative polyketide synthase MbtD	1010	108.2	MAB_2119c
15	B1MCS0	Probable fatty-acid-CoA ligase FadD	1183	128.2	MAB_2963

**Table 14:** Surface proteins unique to secondary biofilm (SCFM).

	Accession	Description	# AAs	MW [kDa]	Ensembl Gene ID
1	B1MCA2	Uncharacterized protein (low molecular weight protein antigen 6 PH domain-containing)	150	16.3	MAB_2794c
2	B1MLN0	Conserved hypothetical transmembrane protein	173	18.3	MAB_1371
3	B1MJY7	Uncharacterized protein (Polyketide cyclase/dehydrase and lipid transport)	202	21.6	MAB_4424
4	B1MDE2	Phosphatidate cytidylyltransferase	292	30.5	MAB_3186c
5	B1MHG6	Possible cytochrome C-type biogenesis protein CcsA	323	35.1	MAB_3973c
6	B1MJE2	Putative MCE family protein	348	38.1	MAB_1008c
7	B1ML21	Putative Mce family protein	346	38.3	MAB_4597c
8	B1MDC6	Probable protease/peptidase	415	44.4	MAB_3170c
9	B1MMW0	Probable fatty acid desaturase	403	46	MAB_1587c
10	B1MP19	Probable serine/threonine protein kinase	434	46.6	MAB_1988c
11	B1MKM5	Uncharacterized protein	465	49.4	MAB_1229
12	B1ME36	Putative integral membrane protein	467	50.7	MAB_3429
13	B1MMK9	Uncharacterized protein	458	51.8	MAB_4926
14	B1MG86	Uncharacterized protein (EccD-like transmembrane domain-containing protein)	511	52.2	MAB_3757
15	B1MHG7	Putative cytochrome C biogenesis protein ResB	535	58.5	MAB_3974c
16	B1MDA0	Putative ABC transporter, ATP-binding protein	652	67.3	MAB_3144
17	B1ME06	Probable ABC transporter, ATP-binding	623	70.4	MAB_3399
18	B1MEP1	Uncharacterized protein (alanine and leucing rich)	662	71.2	MAB_0205c
19	B1MJG5	Divalent metal cation transporter MntH	409	43	MAB_1031c
20	B1MAL6	Apolipoprotein N-acyltransferase	602	64	MAB_2206
21	B1MDK8	Sensor protein KdpD	835	88.6	MAB_3251c
22	B1MEM1	Probable arabinosyltransferase B	1071	116.2	MAB_0185c
23	B1MLS6	Putative lipoprotein LprC	185	19.4	MAB_1417
24	B1MBT2	Branched-chain amino acid ABC transporter (LivG)	314	34.4	MAB_2623c
25	B1MBT2	SGNHhydro domain-containing protein	335	35.1	MAB_1193c
26	B1MIS5	Probable glutamine-binding protein GlnH	327	35.9	MAB_4223
27	B1ML22	Putative Mce family protein	350	37.8	MAB_4598c
28	B1MKW8	Diacylglycerol O-acyltransferase	457	49.5	MAB_4544c

**Table 15:** Surface proteins shared across all treatment groups.

	Accession	Description	# AAs	MW [kDa]	Ensembl Gene ID
1	B1MLV9	ATP synthase subunit b-delta	448	47.6	MAB_1450
2	B1MLW0	ATP synthase subunit alpha	548	58.8	MAB_1451
3	B1MLW3	ATP synthase epsilon chain	121	13.1	MAB_1454
4	B1ME62	Cell wall synthesis protein CwsA	134	13.6	MAB_0024c
5	B1MAP3	Conserved hypothetical membrane protein	521	54.6	MAB_2233c
6	B1MN13	Elongation factor 4	617	68	MAB_1640
7	B1MLW1	ATP synthase gamma chain	308	33.1	MAB_1452
8	B1ME71	Probable serine/threonine-protein kinase PknB	628	67	MAB_0033c
9	B1MH53	Phosphatidylserine decarboxylase proenzyme	237	25	MAB_0639c
10	B1MLW2	ATP synthase subunit beta	476	51.7	MAB_1453
11	B1MG88	Uncharacterized protein (Type VII secretion protein EccB)	493	52	MAB_3759c
12	B1MDJ8	Signal recognition particle receptor FtsY	458	47.8	MAB_3241c
13	B1MF49	Protein translocase subunit SecA	929	103.9	MAB_3580c
14	B1MEY4	Possible transmembrane cation transporter	361	38.6	MAB_3514c
15	B1MF02	Uncharacterized protein	406	42.8	MAB_3532
16	B1MM29	Putative membrane protein, MmpL	965	104.2	MAB_4746
17	B1MB81	PknH_C domain-containing protein	251	26.5	MAB_2421c
18	B1MG27	Putative ABC transporter, ATP-binding protein	861	91.1	MAB_3698
19	B1MEL4	Hypothetical cutinase	336	35.7	MAB_0178
20	B1MNZ2	Probable cytochrome c oxidase subunit 2	349	38.7	MAB_1961
21	B1MCB4	Lipoprotein LprG (27 kDa lipoprotein)	225	23.2	MAB_2806
22	B1MDM6	Thioredoxin-like_fold domain-containing protein	257	27.9	MAB_3269c
23	B1MG04	Succinate dehydrogenase flavoprotein subunit	588	64.8	MAB_3675
24	B1MK36	Uncharacterized protein	393	42.8	MAB_4474
25	B1MC26	PHB domain-containing protein	380	41.1	MAB_2718c
26	B1MAP2	Putative FtsK/SpoIIIE family protein	1343	146.7	MAB_2232c
27	B1MN79	TPM_phosphatase domain-containing protein	671	70.8	MAB_1706c
28	B1MLM3	Probable serine protease HtrA	492	50.6	MAB_1364
29	B1MEE1	Probable oxidoreductase	412	45.3	MAB_0105c
30	B1MMI4	Penicillin-binding protein	793	83.2	MAB_4901c
31	B1MJY5	Putative succinate dehydrogenase, flavoprotein subunit	641	70.1	MAB_4422
32	B1MEL6	Polyketide synthase PKS13	1782	189.7	MAB_0180
33	B1MAM2	Probable polyketide synthase	2121	226.9	MAB_2212
34	B1MGU0	Probable conserved lipoprotein LpqG	249	25.7	MAB_0525c
35	B1MCI6	SBP_bac_5 domain-containing protein	558	58.9	MAB_2878c
36	B1MEV1	Probable acyl-CoA dehydrogenase FadE	582	64.6	MAB_3481
37	B1MLJ1	Probable pyrroline-5-carboxylate dehydrogenase RocA	544	58.6	MAB_1330
38	B1MKD6	Putative polyketide synthase Pks16	549	59	MAB_1140
39	B1MKF8	Uncharacterized protein (Efem/EfeO family lipoprotein)	384	41.3	MAB_1162c
40	B1MJ75	Probable polyketide synthase	3697	393.6	MAB_0939
41	B1MGZ4	Probable acyl-CoA dehydrogenase FadE	715	74.8	MAB_0580
42	B1MCV1	Trk system potassium uptake protein CeoB	229	24.2	MAB_2994
43	B1MP09	Probable long-chain-fatty-acid–CoA ligase FadD	603	63.8	MAB_1978c
44	B1MGG3	Possible L-lactate dehydrogenase (Cytochrome) LldD1	392	41.9	MAB_3834c
45	B1MLD7	Probable fatty-acid-coa ligase FadD	1178	128.6	MAB_4714c
46	B1MJQ1	Probable acyl-CoA dehydrogenase FadE	727	77	MAB_4336
47	B1MD56	Alanine dehydrogenase	371	38.7	MAB_3100
48	B1MK00	Probable acyl-CoA dehydrogenase FadE	614	67.1	MAB_4437
49	B1ML19	Putative Mce family protein	393	40.4	MAB_4595c
50	B1ME12	Putative short chain dehydrogenase/reductase	284	29.6	MAB_3405
51	B1MKE0	Probable short-chain dehydrogenase/reductase	294	31	MAB_1144c
52	B1MG05	Probable succinate dehydrogenase SdhB	260	29	MAB_3676
53	B1MJK2	Uncharacterized protein	200	21.7	MAB_1068c
54	B1MM89	Uncharacterized protein (LysM domain-containing protein)	449	49	MAB_4806c
55	B1MJG0	DUF1942 domain-containing protein (MPT63-like domain)	310	30.1	MAB_1026c
56	B1MKG1	Enolase	430	45	MAB_1165
57	B1MNW5	Glutamine synthetase	478	53.4	MAB_1933c
58	B1MAY1	Translation initiation factor IF-3	176	19.5	MAB_2321
59	B1MG60	60 kDa chaperonin	539	56	MAB_3731c

## Discussion

The intrinsic resistance of *M. abscessus* to available antimicrobials creates a major challenge for the treatment of the disease in high-risk populations (18, 19). Therefore, improving our understanding of the pathogenesis of infection in these populations is needed to develop alternative ways to prevent or treat the infectious condition.

*M. abscessus*, like other environmental microorganisms, has the ability to form biofilms on surfaces, which has been shown as a strategy used by the pathogen to establish a niche on the airway mucosa (12, 20). Past observations in both animal models and humans have demonstrated that *M. abscessus* biofilm is part of the pathogenesis of lung infections (4, 21). Recent studies have determined that *M. abscessus* cultured in SCFM, which mimics the mucus environment in the airways of patients with cystic fibrosis, forms a biofilm that differs from biofilms formed under water or in buffer conditions (22, 23), which indicates that in the presence of sputum contents in the airways *M. abscessus* differentiates and acquires a new phenotype. In addition, the biofilm’s extracellular matrix is formed by eDNA and glycol-phospholipids (21), which, in the case of *Mycobacterium avium*, has been shown to protect the bacteria against the action of phagocytic cells (24).

In this work, we demonstrate that bacteria in *M. abscessus* biofilm at some point are able to release from the initial biofilm mass and probably seed in an adjacent site in the airway. This information is important since in our model it was observed that bacteria in biofilms formed under SCFM sputum conditions, once detached, can establish another biofilm with significantly greater efficiency than bacteria in biofilm developed under phosphate buffer conditions. In fact, previous studies in the laboratory have determined that *M. abscessus* is capable of utilizing the magnesium concentration in the cystic fibrosis medium environment to quickly establish a niche and develop a robust biofilm (9). Furthermore, bacteria released from biofilms in the airways can be expelled in the sputum, which increases the chance of transmission by aerosols in the airway of a second individual. In fact, recent observations by diverse groups have suggested that non-tuberculous mycobacteria can be transmitted from an individual to a recipient, most likely in the environment of clinics that assist patients belonging to at-risk groups (6, 7, 25). The hypotheses and the observations in studies on necropsy in animals or lung transplantation in humans suggest that bacteria released from biofilms may be transported upward or downward depending on the flow of the air either leaving or entering the airways. A limitation of our study is that despite the hypothesis and the previous observations, we do not provide visual proof in this study. A previous study demonstrated the possibility that environmental mycobacteria can be transmitted from person to person, although the prolonged time between infection and disease makes the epidemiological connection very difficult. Using *Caenorhabditis elegans* as a model, *M. avium* infection could be transmitted directly from one host to another without the environmental step that was previously thought to be required (26, 27). A clear consequence of these findings is that an infection can be transmitted from host to host without the environmental step, which makes the bacterial phenotype released from the biofilm one of the potential phenotypes that transmit the infection.

Bacteria released from biofilm in cystic fibrosis medium were shown to be able to establish a secondary biofilm at the same rate as the primary biofilm (**Figure 2**). The SCFM biomass was more robust than the biofilm formed by bacteria released from HBSS-established biofilm, showing that bacteria in the presence of a cystic fibrosis environment can still efficiently seed in another location in the airways. In addition, detached *M. abscessus* invades epithelial cells more efficiently than planktonic bacteria, indicating that the process of binding to establish a primary biofilm and then the subsequent release and formation of a secondary biofilm is associated with bacterial structural modifications, most likely on the bacterial surface. The number of different proteins expressed by the bacterial surface released from biofilms in comparison with bacteria attached to the biofilm is striking. When comparing proteins uniquely expressed by the released bacteria that were not expressed in the other conditions, several uncharacterized proteins, an Mg++ transport, and a few lipoproteins and polyketide synthases were identified. Matching the mutant MAB_3538, which has kinase activity, in the detached biofilm groupings is an interesting finding. A definitive analysis of the function of these proteins is warranted.

Although the changes on the bacterial surface described in this study and in recent publications (22, 23) are responsible for the different interactions between the pathogen and host cells, an increased uptake by the phagocyte could be a plausible outcome. Our results also demonstrated that the ingested bacteria are not killed by macrophages and grow inside the phagocytic cells at a higher rate than planktonic bacteria. The question of why bacteria uptake is increased with better survival outcomes is pertinent here. Since significant changes occur during intracellular survival, one may hypothesize that the presence of adherent molecules on the bacterial surface is advantageous for disease progression. More research needs to be conducted to understand the effects of the identified genes during infections.

The ability of several different bacterial species to detach from biofilms seems to be dictated by a quorum-sensing mechanism (28). Quorum sensing is well-described for Gram-negative bacteria, mostly *Pseudomonas aeruginosa*, Vibrio sp. and Gram-positive bacteria such as *Bacillus cereus* and *Staphylococcus aureus* (28). There are three known quorum-sensing types. Type I utilizes N-acyl homoserine lactone, while the other two (types II and III) are regulated by other autoinducers, many of them peptides (28–30). Autoinducers accumulate in the environment as the bacteria population increases and stimulate the expression of membrane transporters and activation of histidine kinases inside the bacterium. *P. aeruginosa* has several interconnected quorum-sensing circuits that collectively regulate hundreds of genes (31). *Pseudomonas* has three major circuits that regulate approximately 10% of the bacterial genes (32). Two circuits respond to N-acyl homoserine lactone signals and a third one, the *Pseudomonas* quinolone signal system, uses the quinolone signal to interact with RHI receptors which also recognize homoserine lactone signals. These signals are detected by receptors present in the cytoplasm or in the membrane. Gram-positive bacteria, in contrast, use peptides as signaling molecules. They usually bind to membrane histidine kinase receptors that autophosphorylate, although, in some cases, the peptides are transported in the cytoplasm where they interact with the transcription factors (28). Mycobacterial proteins do not share any homology with known quorum-sensing-linked proteins of different bacteria. However, several of the small proteins identified in our work as being expressed under biofilm in SCFM and detachment phenotypes have signal peptides and are potentially exported in the biofilm setting. Other researchers are currently addressing this hypothesis regarding the function of surface proteins and their potential participation as signal proteins (13, 28, 33–35).

One should consider that detachment from biofilms may depend on the type and the status of the patient’s immune system, which can interfere with the dissemination of the pathogen in their lungs. It is also important to take into account the pathogen’s phenotype which can be influenced by the lung environment (36).

In summary, this work shows that *M. abscessus* can release from biofilms on the surface of mucosal epithelial cells and these released bacteria are very efficient in developing a new biofilm to bind to mucosal epithelial cells and infect macrophages. Based on the findings that *M. abscessus* can infect patients directly, the released bacteria can be considered an infectious phenotype. The proteomic sequences of the released bacterial surface proteins identified potential candidates involved in the detachment from biofilm and these need to be studied further.

## Data Availability

The datasets presented in this study can be found in online repositories. The names of the repository/repositories and accession number(s) can be found in the article/supplementary material.

## References

[1] Floto RA Haworth CS. The growing threat of nontuberculous mycobacteria in CF. J Cyst Fibros. (2015) 14:1–2. doi: 10.1016/j.jcf.2014.12.002 25487786

[2] Martiniano SL Nick JA Daley CL. Nontuberculous mycobacterial infections in cystic fibrosis. Clin Chest Med. (2022) 43:697–716. doi: 10.1016/j.ccm.2022.06.010 36344075

[3] Johansen MD Herrmann J-L Kremer L. Non-tuberculous mycobacteria and the rise of Mycobacterium abscessus. 7. Nat Rev Microbiol. (2020) 18:392–407. doi: 10.1038/s41579-020-0331-1 32086501

[4] Ryan K Byrd TF. Mycobacterium abscessus: shapeshifter of the mycobacterial world. Front Microbiol. (2018) 9:2642. doi: 10.3389/fmicb.2018.02642 30443245 PMC6221961

[5] Daley CL Iaccarino JM Lange C Cambau E Wallace RJ Andrejak C. Treatment of nontuberculous mycobacterial pulmonary disease: an official ATS/ERS/ESCMID/IDSA clinical practice guideline. Eur Respir J. (2020) 56:2000535. doi: 10.1183/13993003.00535-2020 32636299 PMC8375621

[6] Bryant JM Brown KP Burbaud S Everall I Belardinelli JM Rodriguez-Rincon D. Stepwise pathogenic evolution of Mycobacterium abscessus. Science. (2021) 372:eabb8699. doi: 10.1126/science.abb8699 33926925 PMC7611193

[7] Ruis C Bryant JM Bell SC Thomson R Davidson RM Hasan NA. Dissemination of Mycobacterium abscessus via global transmission networks. Nat Microbiol. (2021) 6:1279–88. doi: 10.1038/s41564-021-00963-3 PMC8478660 34545208

[8] Miranda-CasoLuengo AA Staunton PM Dinan AM Lohan AJ Loftus BJ. Functional characterization of the Mycobacterium abscessus genome coupled with condition specific transcriptomics reveals conserved molecular strategies for host adaptation and persistence. BMC Genomics. (2016) 17:553. doi: 10.1186/s12864-016-2868-y 27495169 PMC4974804

[9] Keefe BF Bermudez LE. Environment in the lung of cystic fibrosis patients stimulates the expression of biofilm phenotype in Mycobacterium abscessus. J Med Microbiol. (2022) 71:001467. doi: 10.1099/jmm.0.001467 35014948

[10] Fennelly KP Ojano-Dirain C Yang Q Liu L Lu L Progulske-Fox A. Biofilm formation by mycobacterium abscessus in a lung cavity. Am J Respir Crit Care Med. (2016) 193:692–3. doi: 10.1164/rccm.201508-1586IM 26731090

[11] Belardinelli JM Li W Avanzi C Angala SK Lian E Wiersma CJ. Unique features of mycobacterium abscessus biofilms formed in synthetic cystic fibrosis medium. Front Microbiol. (2021) 12:743126. doi: 10.3389/fmicb.2021.743126 34777289 PMC8586431

[12] Rose SJ Babrak LM Bermudez LE. Mycobacterium avium possesses extracellular DNA that contributes to biofilm formation, structural integrity, and tolerance to antibiotics. PloS One. (2015) 10:e0128772. doi: 10.1371/journal.pone.0128772 26010725 PMC4444313

[13] Palmer KL Mashburn LM Singh PK Whiteley M. Cystic fibrosis sputum supports growth and cues key aspects of Pseudomonas aeruginosa physiology. J Bacteriol. (2005) 187:5267–77. doi: 10.1128/JB.187.15.5267-5277.2005 PMC1196007 16030221

[14] Rose SJ Bermudez LE. Identification of bicarbonate as a trigger and genes involved with extracellular DNA export in mycobacterial biofilms. mBio. (2016) 7:e01597–16. doi: 10.1128/mBio.01597-16 PMC5142616 27923918

[15] Leestemaker-Palmer AL Bermudez LE. Mycobacterium abscessus infection results in decrease of oxidative metabolism of lung airways cells and relaxation of the epithelial mucosal tight junctions. Tuberculosis (Edinb). (2023) 138:102303. doi: 10.1016/j.tube.2023.102303 36652813

[16] Lewis MS Danelishvili L Rose SJ Bermudez LE. MAV_4644 Interaction with the Host Cathepsin Z Protects Mycobacterium avium subsp. hominissuis from Rapid Macrophage Killing. Microorganisms. (2019) 7:144. doi: 10.3390/microorganisms7050144 31117286 PMC6560410

[17] Danelishvili L Rojony R Carson KL Palmer AL Rose SJ Bermudez LE. Mycobacterium avium subsp. hominissuis effector MAVA5_06970 promotes rapid apoptosis in secondary-infected macrophages during cell-to-cell spread. Virulence. (2018) 9:1287–300. doi: 10.1080/21505594.2018.1504559 PMC6177253 30134761

[18] Griffith DE Girard WM Wallace RJ. Clinical features of pulmonary disease caused by rapidly growing mycobacteria. An analysis of 154 patients. Am Rev Respir Dis. (1993) 147:1271–8. doi: 10.1164/ajrccm/147.5.1271 8484642

[19] Nessar R Cambau E Reyrat JM Murray A Gicquel B. Mycobacterium abscessus: a new antibiotic nightmare. J Antimicrob Chemother. (2012) 67:810–8. doi: 10.1093/jac/dkr578 22290346

[20] Esteban J García-Coca M. Mycobacterium biofilms. Front Microbiol. (2017) 8:2651. doi: 10.3389/fmicb.2017.02651 29403446 PMC5778855

[21] Blanchard JD Elias V Cipolla D Gonda I Bermudez LE. Effective Treatment of Mycobacterium avium subsp. hominissuis and Mycobacterium abscessus Species Infections in Macrophages, Biofilm, and Mice by Using Liposomal Ciprofloxacin. Antimicrob Agents Chemother. (2018) 62:e00440–18. 10.1128/AAC.00440-18 PMC6153787 30012773

[22] Wiersma CJ Belardinelli JM Avanzi C Angala SK Everall I Angala B. Cell Surface Remodeling of Mycobacterium abscessus under Cystic Fibrosis Airway Growth Conditions. ACS Infect Dis. (2020) 6:2143–54. doi: 10.1021/acsinfecdis.0c00214 32551551

[23] Leestemaker-Palmer A Ong T Bermudez LE. Exposure of Mycobacteriodes abscessus to mucin affects bacterial phenotype. Sci Rep. (2024) 15:393. 10.1038/s41598-024-84451-8 PMC11697318 39747334

[24] Rose SJ Bermudez LE. Mycobacterium avium biofilm attenuates mononuclear phagocyte function by triggering hyperstimulation and apoptosis during early infection. Infect Immun. (2014) 82:405–12. doi: 10.1128/IAI.00820-13 PMC3911830 24191301

[25] Kreutzfeldt KM McAdam PR Claxton P Holmes A Seagar AL Laurenson IF. Molecular longitudinal tracking of Mycobacterium abscessus spp. during chronic infection of the human lung. PloS One. (2013) 8:e63237. 23696800 10.1371/journal.pone.0063237 PMC3655965

[26] Everman JL Ziaie NR Bechler J Bermudez LE. Establishing Caenorhabditis elegans as a model for Mycobacterium avium subspecies hominissuis infection and intestinal colonization. Biol Open. (2015) 4:1330–5. doi: 10.1242/bio.012260 PMC4610217 26405050

[27] Bermudez LE Rose SJ Everman JL Ziaie NR. Establishment of a Host-to-Host Transmission Model for Mycobacterium avium subsp. hominissuis Using Caenorhabditis elegans and Identification of Colonization-Associated Genes. Front Cell Infect Microbiol. (2018) 8:123. doi: 10.3389/fcimb.2018.00123 29740544 PMC5928147

[28] Rutherford ST Bassler BL. Bacterial quorum sensing: its role in virulence and possibilities for its control. Cold Spring Harb Perspect Med. (2012) 2:a012427. doi: 10.1101/cshperspect.a012427 23125205 PMC3543102

[29] Boles BR Horswill AR. Agr-mediated dispersal of Staphylococcus aureus biofilms. PloS Pathog. (2008) 4:e1000052. doi: 10.1371/journal.ppat.1000052 18437240 PMC2329812

[30] Borlee BR Geske GD Blackwell HE Handelsman J. Identification of synthetic inducers and inhibitors of the quorum-sensing regulator LasR in Pseudomonas aeruginosa by high-throughput screening. Appl Environ Microbiol. (2010) 76:8255–8. doi: 10.1128/AEM.00499-10 PMC3008242 20935125

[31] Lazar V. Quorum sensing in biofilms -How to destroy the bacterial citadels or their cohesion/power? Anaerobe. (2011) 117:280–5. 10.1016/j.anaerobe.2011.03.023 21497662

[32] Mattman ME Blackwell HE. Small molecules that modulate quorum sensing and control virulence in Pseudomonas aeruginosa. J Org Chem. (2010) 75:6737–6746. doi: 10.1021/jo101237e 20672805 PMC2952040

[33] Ji G Beavis RC Novick RP. Cell density control of staphylococcal virulence mediated by an octapeptide pheromone. Proc Natl Acad Sci USA. (1995) 92:12055–9. doi: 10.1073/pnas.92.26.12055 PMC40295 8618843

[34] Magnuson R Solomon J Grossman AD. Biochemical and genetic characterization of a competence pheromone from B. subtilis. Cell. (1994) 77:207–16. doi: 10.1016/0092-8674(94)90313-1 8168130

[35] Lilley BN Bassler BL. Regulation of quorum sensing in Vibrio harveyi by LuxO and sigma-54. Mol Microbiol. (2000) 36:940–54. doi: 10.1046/j.1365-2958.2000.01913.x 10844680

[36] Leestemaker-Palmer A Ong T Bermudez LE. Environmental conditions encountered in the lung are associated with changes in *Mycobacterioides abscessus* phenotypes over time. Sci Rep. (2025) 15:393. doi: 10.1038/s41598-024-84451-8 39747334 PMC11697318

